# Nonstop mRNAs generate a ground state of mitochondrial gene expression noise

**DOI:** 10.1126/sciadv.abq5234

**Published:** 2022-11-18

**Authors:** Kah Ying Ng, Guleycan Lutfullahoglu Bal, Uwe Richter, Omid Safronov, Lars Paulin, Cory D. Dunn, Ville O. Paavilainen, Julie Richer, William G. Newman, Robert W. Taylor, Brendan J. Battersby

**Affiliations:** ^1^ Institute of Biotechnology, Helsinki Institute of Life Science, University of Helsinki, Helsinki, Finland.; ^2^ Faculty of Biological and Environmental Sciences, University of Helsinki, Helsinki, Finland.; ^3^ Wellcome Centre for Mitochondrial Research, Biosciences Institute, Faculty of Medical Sciences, Newcastle University, Newcastle upon Tyne, UK.; ^4^ DNA Sequencing and Genomics Laboratory, University of Helsinki, Helsinki, Finland.; ^5^ Department of Medical Genetics, Children’s Hospital of Eastern Ontario, Ottawa, ON, Canada.; ^6^ Manchester Centre for Genomic Medicine, St. Mary’s Hospital, Manchester University NHS Foundation Trust, Manchester, UK.; ^7^ Division of Evolution, Infection and Genomics, School of Biological Sciences, University of Manchester, Manchester, UK.; ^8^ Wellcome Centre for Mitochondrial Research, Translational and Clinical Research Institute, Faculty of Medical Sciences, Newcastle University, Newcastle upon Tyne, UK.; ^9^ NHS Highly Specialised Service for Rare Mitochondrial Disorders, Newcastle upon Tyne Hospitals NHS Foundation Trust, Newcastle upon Tyne, UK.

## Abstract

A stop codon within the mRNA facilitates coordinated termination of protein synthesis, releasing the nascent polypeptide from the ribosome. This essential step in gene expression is impeded with transcripts lacking a stop codon, generating nonstop ribosome complexes. Here, we use deep sequencing to investigate sources of nonstop mRNAs generated from the human mitochondrial genome. We identify diverse types of nonstop mRNAs on mitochondrial ribosomes that are resistant to translation termination by canonical release factors. Failure to resolve these aberrations by the mitochondrial release factor in rescue (MTRFR) imparts a negative regulatory effect on protein synthesis that is associated with human disease. Our findings reveal a source of underlying noise in mitochondrial gene expression and the importance of responsive ribosome quality control mechanisms for cell fitness and human health.

## Teaser

Deep sequencing analysis of the mitochondrial transcriptome identifies sources of non-stop mRNAs and role of MTRFR.

## INTRODUCTION

Across all domains of life, responsive quality control mechanisms have evolved to resolve gene expression errors to maintain cell fitness (*1*, *2*). A potent source of mistakes can arise from failures to terminate protein synthesis. This step is traditionally understood when a translating ribosome encounters a stop codon within the mRNA, which is then recognized by a release factor that catalyzes release of the nascent polypeptide from the transfer RNA (tRNA). In contrast, translation of an mRNA missing a stop codon impedes the canonical mechanisms for translation termination and thereby generates a nonstop ribosome complex (*1*, *2*). A key question remains: How frequently does this type of mRNA arise? We addressed this question for human mitochondrial gene expression. Impairments in mitochondrial protein synthesis are associated with human pathologies that manifest with exceptional cell- and tissue-specific consequences, differing in age of onset and severity (*3*). Thus, elucidating the molecular basis by which defects in mitochondrial protein synthesis can arise is critical to understanding the molecular pathogenesis of these disorders.

**Fig. 1.**
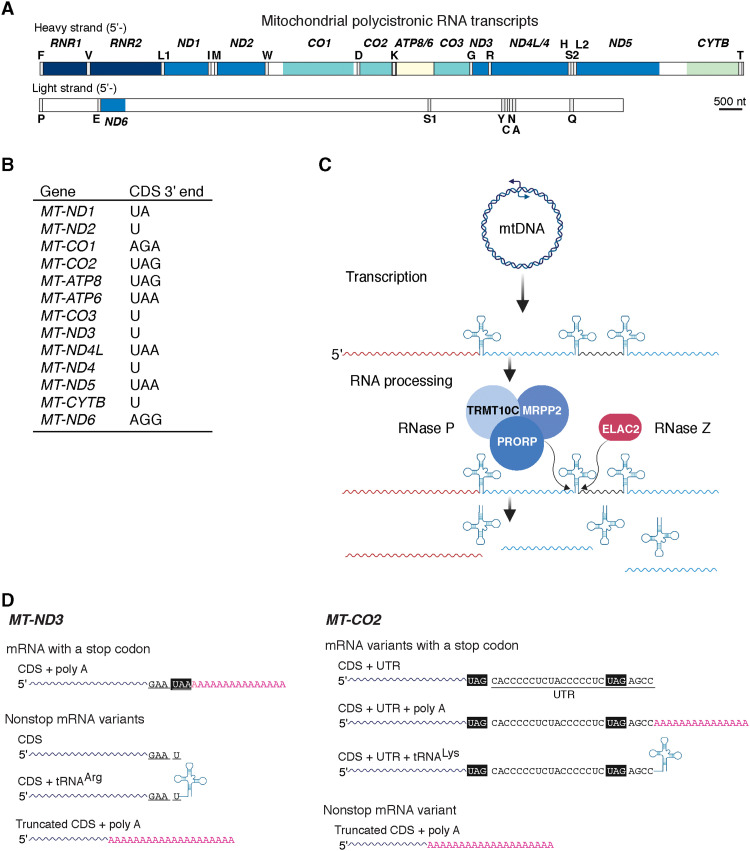
Mitochondrial RNA processing and generation of nonstop mRNA. (**A**) Schematic of the polycistronic RNA transcribed from the heavy- and light-strand promoters in human mtDNA. (**B**) The 3′ end of the human mitochondrial mRNA coding sequences (CDSs). (**C**) Schematic of mitochondrial RNA processing according to the punctuation model whereby tRNAs act as beacons for the indicated nucleases to cleave at the 5′ and 3′, thus liberating individual tRNAs, mRNAs, and rRNAs. Image created with BioRender.com. (**D**) Schematic example of representative potential mitochondrial mRNAs following RNA processing. Bottom left: *MT-ND3* does not encode a stop codon within the CDS and has a tRNA directly flanking the 3′ end. Polyadenylation of the transcript will generate a stop codon (CDS + poly A). All other transcripts are potential nonstop mRNA variants. Bottom right: *MT-CO2* encodes a stop codon and has a short 3′ untranslated region (3′UTR). Image created with BioRender.com.

Human mitochondrial DNA (mtDNA) is one of the most simplified cellular genomes (*4*), encoding only 13 mRNAs that are translated into hydrophobic membrane protein subunits of the oxidative phosphorylation complexes (*5*). Transcription generates long polycistronic messages where 8 of the 13 mitochondrial mRNAs do not encode a standard stop codon (UAA or UAG), and 6 of these have a tRNA directly flanking the 3′ end (Fig. 1, A and B) (*4*). The encoded tRNAs facilitate 5′ and 3′ endonucleolytic processing by a mitochondrial ribonuclease P (RNase P) and RNase Z, respectively, to liberate individual mRNAs and ribosomal RNAs (rRNAs) (Fig. 1C) (*6*, *7*). To reconcile this, the general understanding has been that these six mRNAs require polyadenylation to generate a stop codon (Fig. 1D) (*8*). Thus, half of the mitochondrial mRNAs require precise RNA cleavage followed by posttranscriptional adenylation to generate transcripts with a functional stop codon. Disruptions of either process could potentially form a potent source of mitochondrial nonstop mRNAs.

Using a next-generation sequencing approach with single-nucleotide resolution, we investigated the fidelity of these processes in health and disease. Our findings establish steady-state levels of different types of mitochondrial nonstop mRNAs across healthy human cell types and skeletal muscle. Further, we show that this abundance selectively increases with pathogenic variants that impair mitochondrial protein synthesis and manifest as distinct clinical entities. Last, we demonstrate the functionality of the mitochondrial release factor in rescue (MTRFR) as a ribosome quality control factor for resolving aberrations in mitochondrial protein synthesis arising from the translation of nonstop mRNA. Collectively, our study broadens the basic understanding of mitochondrial gene expression while simultaneously revealing a novel molecular mechanism for human mitochondrial pathologies.

## RESULTS

To investigate the frequency of mitochondrial nonstop mRNAs, we used the highly processive group II intron reverse transcriptase (TGIRT) for template switching complementary DNA (cDNA) synthesis (*9*) to capture the 3′ nucleotide (nt) of the mRNAs for deep sequencing (figs. S1 and S2). We sampled the mRNAs associated with mitochondrial ribosomes and the whole mitochondrial transcriptome to distinguish translation regulation. This strategy appears to be a more accurate indicator of mRNAs used to synthesize the oxidative phosphorylation subunits (fig. S1, A to C) (*10*–*12*). Together, our approach allows the unbiased capture of the 3′ nt and comprehensive coverage of the entire mitochondrial transcriptome (fig. S1), including those of highly structured tRNAs (*13*). We chose this approach over ribosome profiling (*14*) because the intrinsic requirement for a ribonuclease treatment introduces a technical bias when analyzing the 3′ end of transcripts not protected by the ribosome. Thus, we can accurately determine the fidelity of RNA processing and posttranscriptional polyadenylation for the six mRNAs (e.g., *ND1*, *ND2*, *CO3*, *ND3*, *ND4*, and *CYTB*) that do not encode stop codons and abut a flanking tRNA sequence at the 3′ end (Fig. 1, A and B).

### Pervasive generation of mitochondrial nonstop mRNAs

**Fig. 2.**
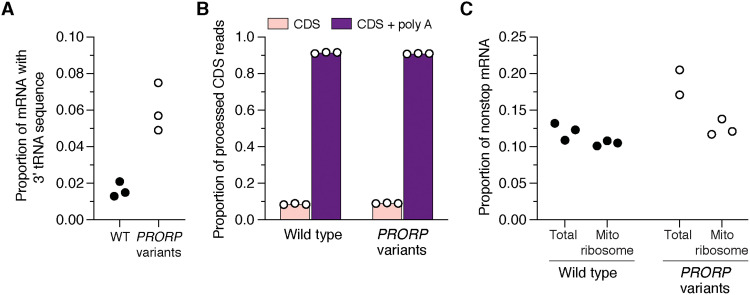
Mitochondrial nonstop mRNAs are generated in health and disease. (**A** and **B**) Deep sequencing analysis of the 3′ end of the mitochondrial mRNAs *MT-CO3*, *CYTB*, *ND1*, *ND2*, *ND3*, and *ND4* that do not encode a stop codon and require tRNA processing followed by polyadenylation to generate a stop codon. RNA isolated from mitochondrial ribosomes in cultured fibroblasts with the indicated genotypes. CDS + poly A, polyadenylated CDS. WT, wild type. (**C**) Total abundance of mitochondrial nonstop mRNAs sequenced from *MT-CO3*, *CYTB*, *ND1*, *ND2*, *ND3*, and *ND4* transcripts in the total transcriptome versus isolated from mitochondrial ribosomes. Each data point represents an independent sample and library preparations.

**Fig. 3.**
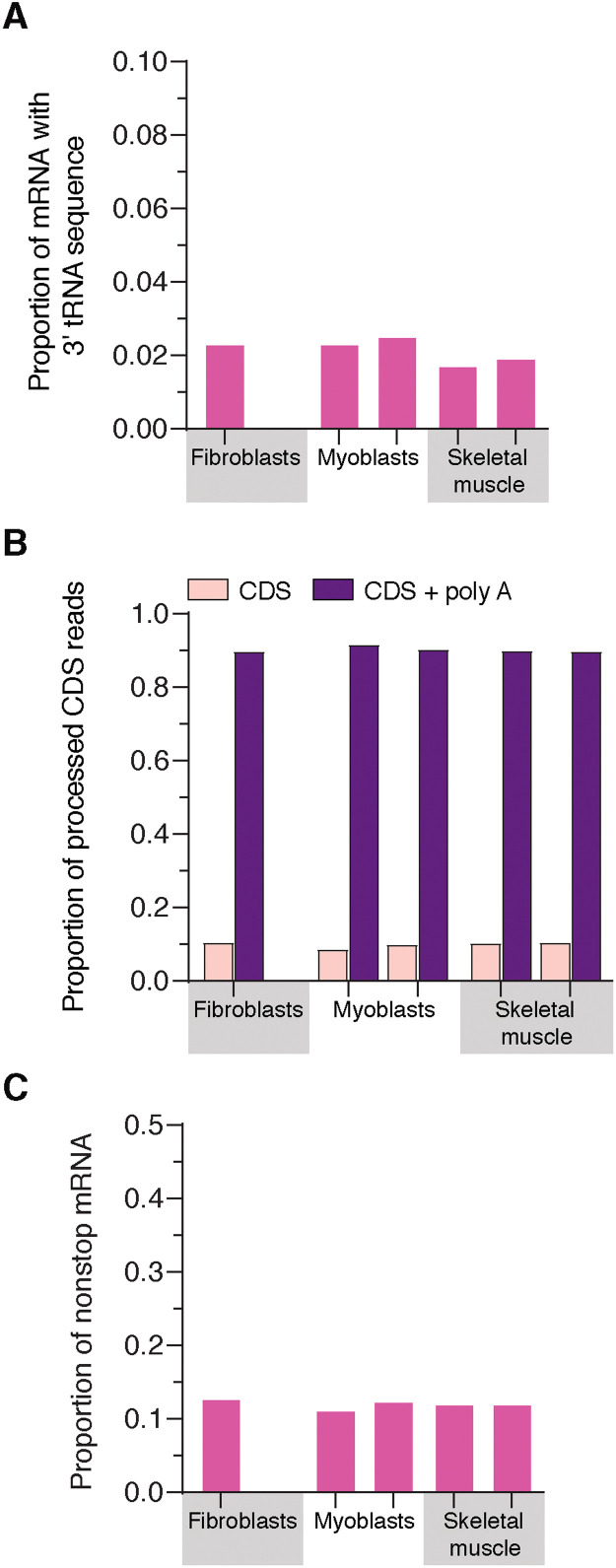
Mitochondrial nonstop mRNAs are generated across healthy cultured cell types and tissues. (**A** to **C**) Deep sequencing analysis of the 3′ end of the mitochondrial mRNAs *MT-CO3*, *CYTB*, *ND1*, *ND2*, *ND3*, and *ND4* in RNA isolated from the indicated cell types and tissue. Each sample is from the whole transcriptome isolated from healthy unrelated individuals. Data are pooled from two independent samples and library preparations.

We first addressed the prevalence of mitochondrial mRNAs that could contain flanking tRNA sequence at the 3′ end. As a positive control, we used patient-derived fibroblasts harboring pathogenic biallelic variants in *PRORP,* encoding the catalytic subunit of the RNase P complex required for 5′ tRNA processing (Fig. 1B and fig. S3) (*6*). These cells demonstrate impaired RNA processing (*15*) but no effect on mitochondrial ribosome assembly (fig. S3). This is in contrast to RNA interference approaches or gene deletions, which generate a profound defect in RNA processing and ribosome assembly and are lethal (*7*, *16*, *17*). In the fibroblasts with pathogenic *PRORP* variants, we identified mRNAs containing flanking tRNA sequence associated with mitochondrial ribosomes (Fig. 2A). Equally important, we also detected a low level of these transcripts in control fibroblasts (Fig. 2A) and wild-type human myoblasts and skeletal muscle (Fig. 3A). Together, these findings demonstrate a baseline level of mitochondrial mRNAs with aberrant 3′ ends across healthy cell types and tissue.

**Fig. 4.**
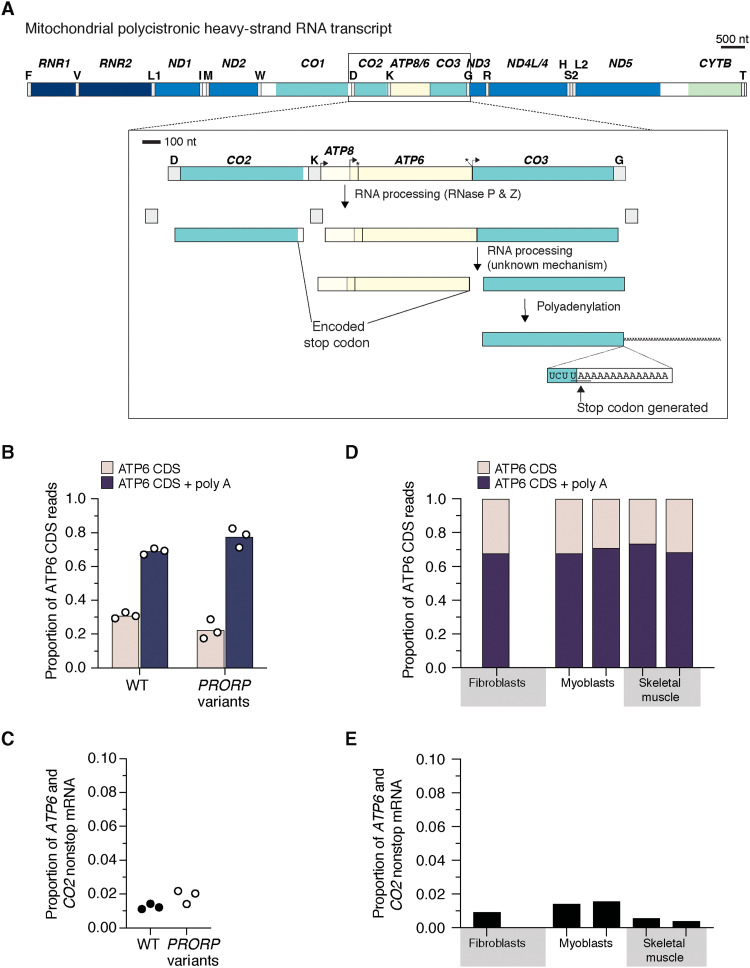
Mitochondrial RNA processing and polyadenlyation for *MT-ATP6*. (**A**) Schematic of the polycistronic heavy-strand RNA transcript highlighting the RNA processing for *MT-CO2*, *ATP6*, and *CO3*. The processing between the ATP6 and CO3 CDS is not mediated by either RNase P or Z. The mechanism of this catalysis is currently unknown. *ATP6* and *CO2* both encode a stop codon in the CDS. (**B**) Deep sequencing analysis of the 3′ end of the processed *ATP6* mRNA from cultured fibroblasts with the indicated genotypes. (**C**) Total nonstop mRNAs for *ATP6* and *CO2* generated by truncated polyadenylated transcripts. (**D**) Deep sequencing analysis of the 3′ end of the processed *ATP6* mRNA from biologically independent healthy controls taken from cultured fibroblasts and myoblasts and human skeletal muscle biopsies. (**E**) Total nonstop mRNAs for *ATP6* and *CO2* generated by truncated polyadenylated transcripts from the samples in (D).

Next, we analyzed the 3′ end of mRNAs correctly processed as the coding sequence (CDS) to determine the frequency of polyadenylation required to generate a stop codon. Unexpectedly, a subset of these mRNAs consistently lack polyadenylation across all cell types investigated (Figs. 2B and 3B). This level was not altered in the fibroblasts with pathogenic *PRORP* variants (Fig. 2B). Variability in the level of polyadenylation was also identified for *MT-ATP6*, which undergoes RNA processing by a PRORP-independent mechanism but encodes a stop codon in the CDS (Figs. 1C and 4, A and B). Furthermore, we detected truncated transcripts with polyadenylation associated with mitochondrial ribosomes, which have been noted before but were considered targets of RNA quality control mechanisms (*18*). Our approach identified three sources of nonstop mitochondrial mRNAs: mRNAs with flanking 3′ tRNA sequence, mRNAs correctly processed but lacking polyadenylation, and truncated polyadenylated transcripts (Figs. 2 to 4). The abundance of nonstop mRNAs appears to differ when comparing mRNAs isolated from mitochondrial ribosomes to the whole transcriptome in the pathogenic *PRORP* variants (Fig. 2C). In addition, translation of mRNAs with a flanking 3′ tRNA sequence or truncated transcripts with polyadenlyation will generate fusion open reading frames (ORFs) with a propensity to misfold as proteins following synthesis and will require proteolytic degradation, potentially as a cotranslational event. Together, our analyses identify a low-level source of mitochondrial gene expression errors that require responsive quality control mechanisms to resolve.

**Fig. 5.**
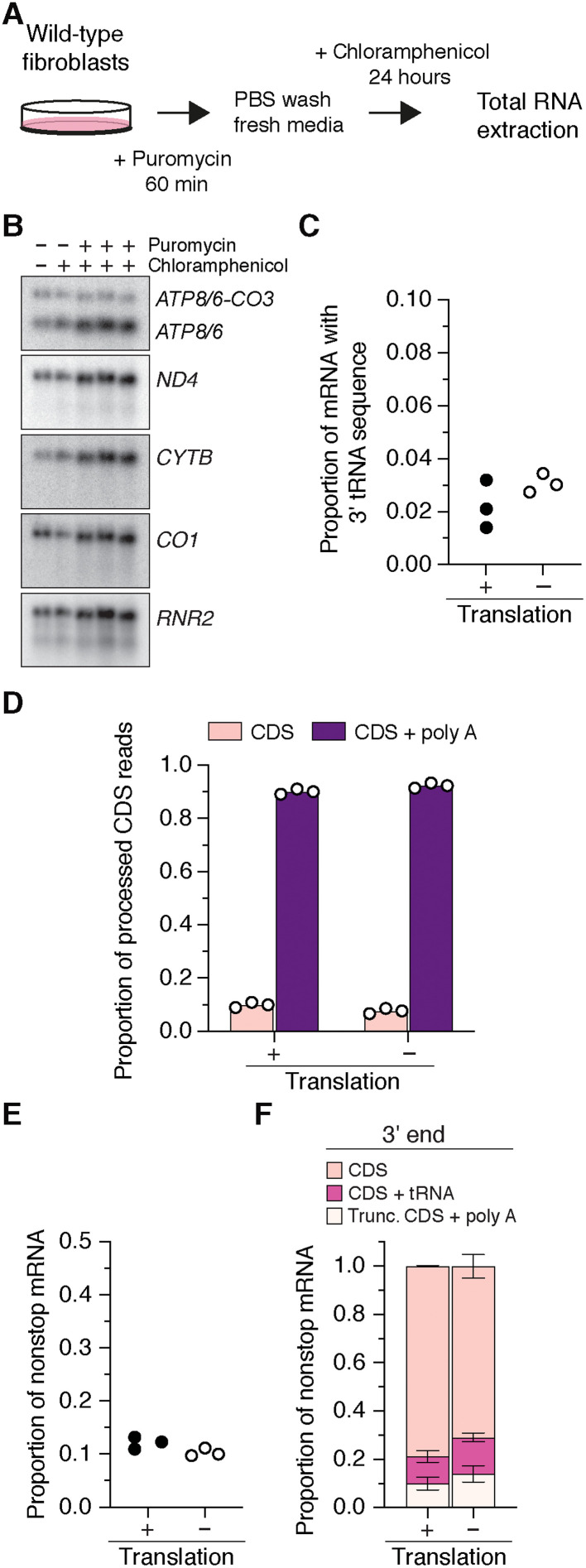
Mitochondrial nonstop mRNAs are generated independent of mitochondrial protein synthesis. (**A**) Workflow to inhibit mitochondrial protein synthesis in cultured wild-type fibroblasts to generate RNA for deep sequencing. PBS, phosphate-buffered saline. (**B**) Northern blotting of total RNA from the whole transcriptome with strand-specific oligonucleotide probes for the indicated RNA transcripts. (**C** to **E**) Deep sequencing of the 3′ end of *MT-CO3*, *CYTB*, *ND1*, *ND2*, *ND3*, and *ND4* from cultured control human fibroblasts with and without mitochondrial translation. CDS + tRNA, CDS with tRNA sequence at the 3′ end; Trunc. CDS + poly A, truncated CDS that were polyadenylated. Each data point represents an independent sample and library preparations. (**F**) Distribution of the types of nonstop mRNAs for *MT-CO3*, *CYTB*, *ND1*, *ND2*, *ND3*, and *ND4*. Data represent means ± SD from three independent samples and library preparations.

To test whether the abundance of these nonstop mRNAs was dependent on mitochondrial protein synthesis, we treated wild-type fibroblasts with puromycin to terminate protein synthesis followed by a 24-hour incubation with chloramphenicol to inhibit translation elongation on mitochondrial ribosomes (Fig. 5A). There was no reduction in the steady-state level of mitochondrial RNA transcripts following this manipulation (Fig. 5B), although mitochondrial RNAs are metabolically unstable in proliferating cells (*19*). Deep sequencing of the RNA showed that the abundance and types of nonstop mRNAs did not change (Fig. 5, C to F). Our data would then suggest that a basal level of mitochondrial nonstop mRNAs and fusion ORFs is consistently generated and that the turnover of these RNAs is independent of protein synthesis. This underscores the importance of posttranscriptional events in mitochondrial RNA metabolism to the fidelity of gene expression.

### Nonstop mRNAs act as negative regulators of protein synthesis

**Fig. 6.**
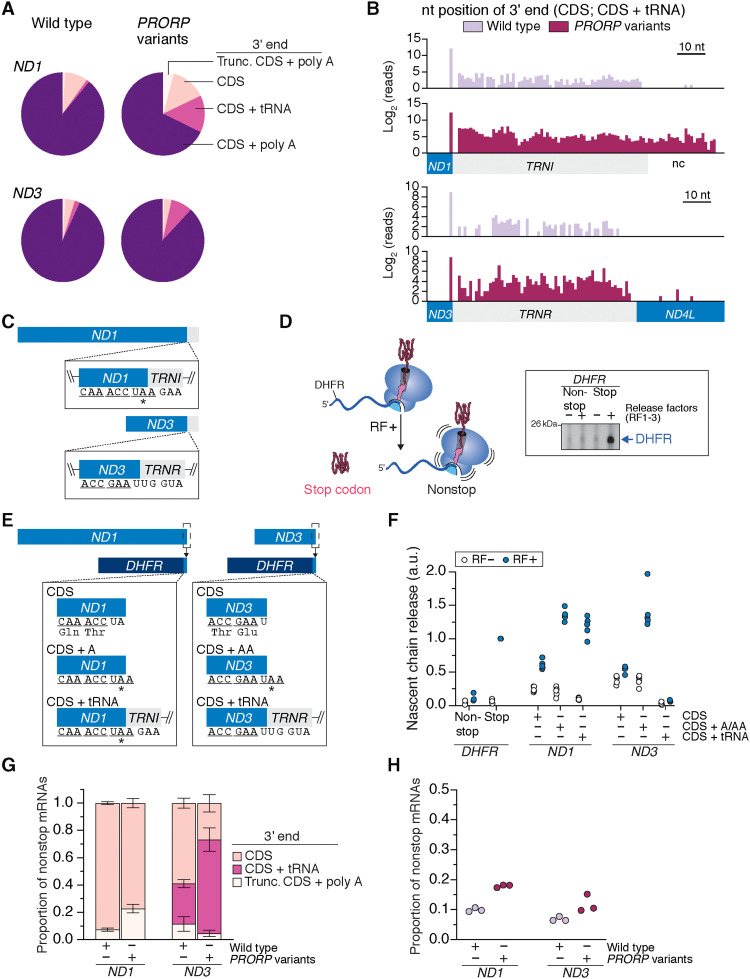
Mitochondrial nonstop mRNAs impair translation termination and act as a negative regulator of protein synthesis. (**A**) Frequency distribution of the 3′ end of mRNAs deep sequenced from mitochondrial ribosomes. (**B**) Frequency histograms of the mapped 3′ nt position of mRNAs on the mitochondrial ribosome not polyadenylated. nc, noncoding. (**C**) Schematic of mRNAs with flanking tRNA sequence; *in-frame stop codon. (**D**) In vitro translation termination assay with *DHFR* as the mRNA template. Release factors (RF) 1 to 3. (**E**) Schematic of chimeric *DHFR*-*ND1* or *ND3* transcripts. (**F**) In vitro translation termination assay using the indicated mRNA templates from (D). (**G**) Nonstop mRNAs for transcripts deep sequenced from mitochondrial ribosomes of cultured human fibroblasts with the indicated genotypes. Means ± SD, *n* = 3. (**H**) Total nonstop mRNAs sequenced from mitochondrial ribosomes. a.u., arbitrary units.

Next, we investigated the effect that translation of nonstop mRNAs exerts on protein synthesis. A central question we sought to address was whether a flanking 3′ tRNA sequence in the mRNA would impede protein synthesis termination, the rationale being that mRNA secondary structures, such as stem loops and pseudoknots, can slow down translation by the ribosome (*20*). We focused on *MT-ND1* and *MT-ND3* as examples (Fig. 6A). These mRNAs encode subunits of the membrane arm of complex I (*21*). Analysis of the mapped 3′ nt position of the tRNA fragment in the CDS + tRNA reads indicates that these are not processing intermediates, as one would predict the full-length tRNA sequence in such a scenario (Fig. 6B). Although *MT-ND1* does not encode a stop codon within the CDS, the *TRNI* fragment generates an in-frame stop codon without extending the length of the nascent polypeptide chain (Fig. 6C). In contrast, the *TRNR* flanking *MT-ND3* does not (Fig. 6C).

We turned to an in vitro biochemical assay to investigate how the types of nonstop mitochondrial mRNAs we identified affect protein synthesis. Since a completely reconstituted mitochondrial translation system has not been established for mitochondrial ribosomes because of the need for cotranslational insertion into the lipid bilayer (*22*), we took advantage of the bacterial Protein synthesis Using Recombinant Elements (PURE) translation system because of the similarities between mitochondrial and bacterial protein synthesis (*5*). In this assay, the absence of a stop codon prevents termination and release of the nascent chain (Fig. 6D). A caveat to this experimental approach and interpretation is that translation on mitochondrial ribosomes could differ. However, this is currently not possible to test in an in vitro system.

Because MT-ND1 and MT-ND3 are polytopic hydrophobic proteins (*21*), translation of the entire ORF in an in vitro assay would lead to protein aggregation, confounding any interpretation. Therefore, we created chimeric mRNA templates fusing the last two codons of *MT-ND1* or *MT-ND3* along with the 3′ sequence variations to the full-length *DHFR* (Fig. 6E). These codon positions are known to affect protein synthesis termination (*23*). In our CDS + tRNA constructs, we took the longest tRNA sequencing read in wild-type cells for both *MT-ND1* and *MT-ND3* (Fig. 6B), fusing them to *DHFR*. Mitochondrial mRNA 3′ ends without a stop codon induced a robust inhibition of nascent chain release (Fig. 6F), whereas a flanking tRNA sequence had differential consequences for translation termination. In the case of *MT-ND1,* the in-frame stop codon encoded with the *TRNI* fragment facilitates termination of protein synthesis (Fig. 6F). In contrast, the *TRNR* flanking *MT-ND3* robustly inhibits the termination of protein synthesis (Fig. 6F). As a result, we consider *MT-ND1* transcripts with flanking tRNA fragments to encode a stop codon. This then restricts *MT-ND1* nonstop mRNAs to two types: processed CDS but lacking polyadenylation and truncated transcripts (Fig. 6, G and H). Similarly, an in-frame stop codon is generated between human *MT-ND2* and the flanking *TRNW* fragment. Our summary analysis of mitochondrial nonstop mRNAs (Figs. 2, 3, and 5) accounts for these findings.

### Mitochondrial AGA and AGG codons will generate nonstop ribosome complexes

**Fig. 7.**
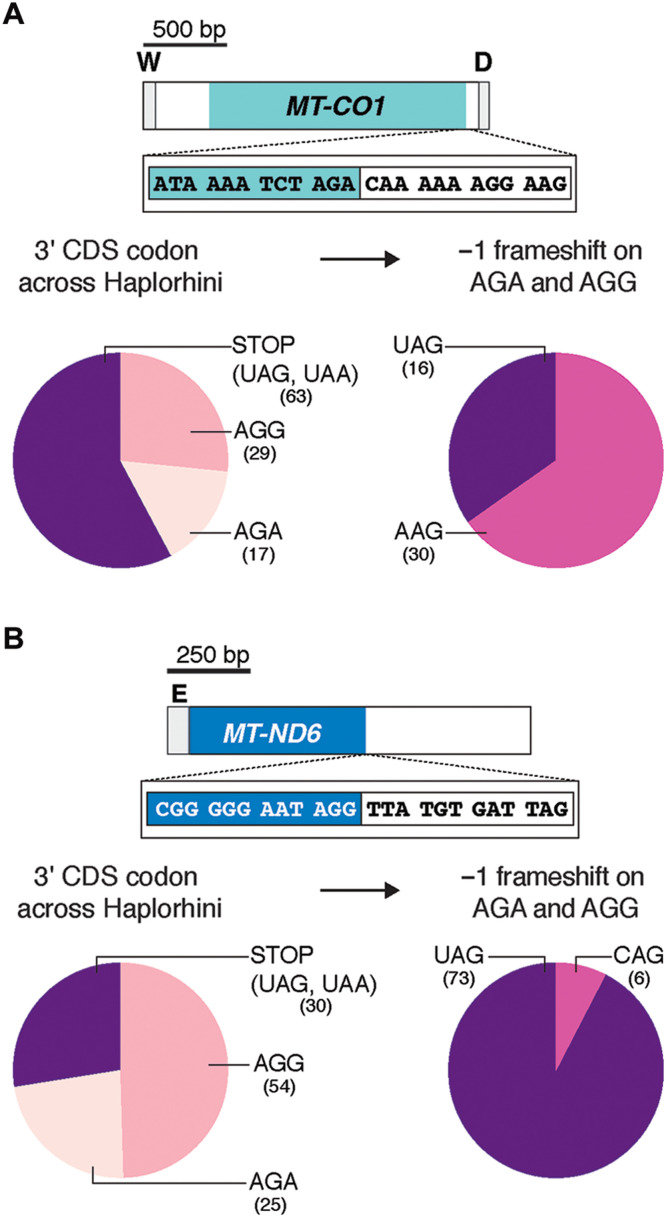
Mitochondrial AGA and AGG act as nonstop codons in gene expression. (**A**) Top: Schematic of the human *MT-CO1* and the 3′ DNA sequence. Bottom left: Analysis of the 3′ codon in the CDS of *MT-CO1* from 109 primate species of the suborder Haplorhini. Bottom right: The predicted 3′ codon following a −1 ribosome frameshift on the AGA and AGG codons. (**B**) The same analysis as in (A) for *MT-ND6*.

One of the peculiarities of the mitochondrial genome is deviation from the universal genetic code (*4*). The AGA and AGG codons found at the 3′ end of the human *MT-CO1* and *MT-ND6* CDS (Figs. 1A and 7 and table S1), respectively, are not recognized by a cognate tRNA. As a result, these two codons have been considered mitochondrial-specific stop codons. At the structural level, however, neither of the two known mitochondrial release factors (MTRF1L and MTRF1) can recognize these codons (*24*, *25*). Meanwhile, an alternative hypothesis proposed that in humans, a −1 frameshift by the mitochondrial ribosome could generate a standard stop codon (UAG) with an upstream U (*26*). A prior phylogenetic analysis of vertebrates, however, suggested that such a frameshifting mechanism was not conserved in mitochondrial genomes, although the study did not rule out a human exception (*27*). If such frameshifting mechanism was the predominant mode for protein synthesis termination on these codons in humans, then one would predict evolutionary conservation of the surrounding sequence among primates to generate the UAG stop codon.

To test this hypothesis, we analyzed the mitochondrial genome sequences of 109 primates from the suborder Haplorhini (Fig. 7 and table S1), which includes humans. Our analysis robustly demonstrates that a potential −1 frameshift does not consistently generate a stop codon among these primates for either AGA or AGG (Fig. 7). The lack of evolutionary conservation, therefore, does not support a strict −1 frameshift mechanism to facilitate protein synthesis termination on these codons. This then begs the question, How could protein synthesis terminate on *MT-CO1* and *MT-ND6* mRNA?

### Resolving termination errors with the rescue factor MTRFR

**Fig. 8.**
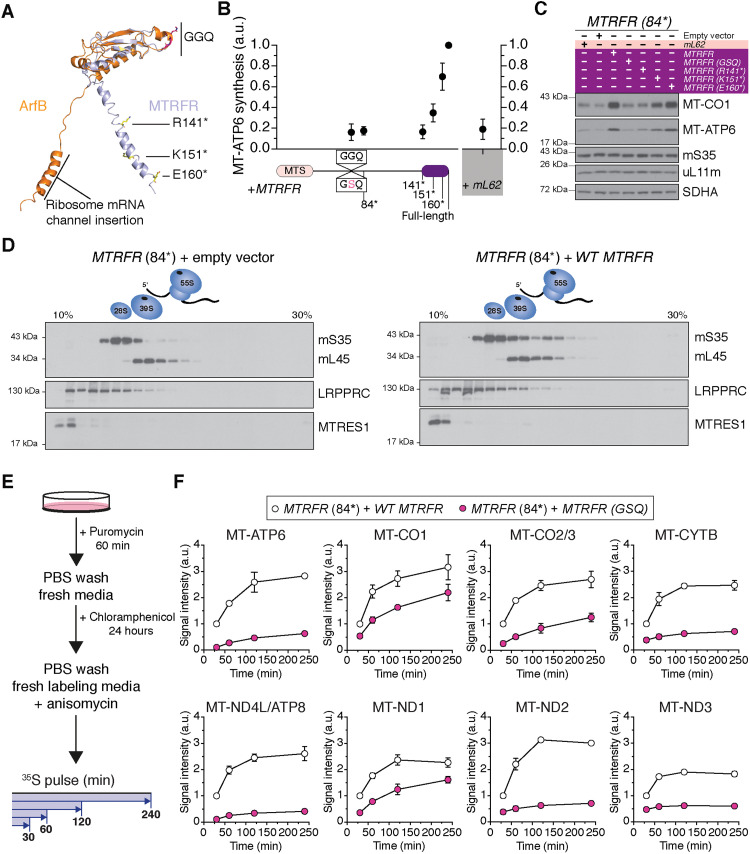
Functional characterization of MTRFR in mitochondrial protein synthesis. (**A**) Structural overlay of MTRFR (C12orf65) [Protein Data Bank (PDB) 7A5H] (*35*) with ArfB (PDB 7JSS) (*32*). The catalytic GGQ motif of the release factors and the position of truncation mutations in MTRFR are indicated. (**B**) ^35^S-metabolic labeling of MT-ATP6 synthesis in MTRFR-deficient (84*) fibroblasts stably transduced with cDNAs of the indicated MTRFR alleles and wild-type *mL62 (ICT1).* Data represent the means ± SD from four independent experiments. MTS, mitochondrial targeting sequence; GGQ, glycine-glycine-glutamine catalytic domain of the release factor. (**C**) Representative immunoblotting of whole cell lysates from (B). (**D**) Immunoblotting of fractions isolated from sucrose density gradient separation of mitochondrial ribosomes from human patient fibroblasts carrying biallelic frameshift variants that terminates MTRFR at codon 84, rendering the protein functionally null. Cells were stably transduced with an empty retroviral vector or a wild-type cDNA of *MTRFR*. The data are representative of multiple independent experiments. (**E**) Schematic of workflow for metabolic labeling of mitochondrial protein synthesis with ^35^S-methionine/cysteine in human patient fibroblasts from (B). (**F**) Quantification of mitochondrial protein synthesis over an extended pulse labeling period. Data represent the means ± SD from three independent experiments.

In the absence of a stop codon, translation termination requires a nonstop rescue mechanism. Mitochondria have two class I release factors (C12orf65/MTRFR and ICT1/mL62) that are structurally homologous to the bacterial alternative ribosome-rescue factor B (ArfB) (Fig. 8A) (*27*–*30*). ArfB has a catalytic GGQ motif but lacks the structural domains to recognize stop codons and is required to terminate protein synthesis on truncated mRNAs (*28*, *31*, *32*). In humans, biallelic stop-gain pathogenic variants in *MTRFR* lead to a progressive neuromuscular presentation (*33*), often with childhood onset, with impaired mitochondrial protein synthesis (Fig. 8, B to D, and fig. S4). Previously, we have shown that MTRFR requires the highly conserved catalytic GGQ motif to resolve the protein synthesis defect (Fig. 8, B and C) (*34*). A structure of a split mitochondrial ribosome revealed MTRFR interacting with the RNA binding protein C6orf203/MTRES1 on the large subunit (*35*). In this structure, however, the GGQ motif was too distal from the ribosome peptidyl transferase center (PTC) to catalyze release of the nascent chain. Further, MTRES1 is not constitutively associated with mitochondrial ribosomes nor is it recruited with MTRFR deficiency (Fig. 8D), and it also appears to bind several different types of mitochondrial RNA (*36*, *37*). In contrast, mL62 is a structural component of the human mitochondrial ribosome located too distal from the PTC to be functional in protein synthesis termination (*38*, *39*), and overexpression does not appear to rescue MTRFR deficiency in patient fibroblasts (Fig. 8, B and C, and fig. S4). Thus, the model proposed for MTRFR function based on the recent cryo–electron microscopy structure (*35*) is unexpectedly at odds with that established for ArfB (*28*, *31*, *32*, *40*). How then could MTRFR terminate protein synthesis mechanistically?

Considering the compelling homology between MTRFR and ArfB (Fig. 8A), we tested whether the mitochondrial release factor functions in an analogous mechanism to the bacterial version. ArfB is recruited to the ribosome translating truncated mRNAs no longer than 9 nt from the decoding center in the ribosome A site (*28*, *31*, *32*). Mechanistically, the ArfB C-terminal α helix inserts into the ribosomal mRNA channel, which repositions the GGQ motif to the PTC to catalyze cleavage of the nascent polypeptide chain from the tRNA (*31*, *32*, *40*). To test the importance of the C-terminal α helix for the MTRFR function in mitochondrial protein synthesis, we generated an allelic series of mutations and stably transduced them into patient-derived fibroblasts that are functionally null for MTRFR (*33*). The data demonstrate a robust genotype-phenotype correlation between the length of the C-terminal α helix and the ability to rescue the defect in mitochondrial protein synthesis and increase the steady-state abundance of individual subunits (Fig. 8, B and C, and fig. S4). The minimal length of the MTRFR C terminus required functionally in protein synthesis overlaps spatially with that of ArfB needed for insertion into the mRNA channel (Fig. 8, A to C).

However, MTRFR deficiency impairs the synthesis of all 13 proteins by mitochondrial ribosomes (fig. S4) (*33*, *34*). This suggests a general effect on mitochondrial protein synthesis rather than a selective one. Our immunoblotting of the steady-state abundance of mitochondrial ribosomal subunits shows that there is not an assembly defect in either the small or large ribosomal subunits with MTRFR deficiency (Fig. 8C). Although isokinetic sucrose gradient separation of mitochondrial ribosomes points to a decrease in the abundance of the 55S monosome (Fig. 8D). This correlates with the reduced metabolic labeling of mitochondrial protein synthesis (Fig. 8B and fig. S4). If our working model is that MTRFR would be required to terminate protein synthesis on nonstop mRNAs, how then would an overall decrease in mitochondrial protein synthesis be generated?

We hypothesize that the defect in mitochondrial protein synthesis with MTRFR deficiency arises as a negative feedback response from the accumulative impairment in protein synthesis termination. Recent mitochondrial ribosome profiling data for human proliferating cultured cells (e.g., fibroblasts and myoblasts) indicate that there are robust differences in the synthesis rates for the 13 mitochondrial proteins (*41*). Thus, the frequency by which a selected mRNA is translated and the inherent propensity of that transcript to be missing a stop codon would be contributing factors to generating nonstop ribosome complexes in mitochondrial gene expression.

To test whether the synthesis of individual proteins is differentially affected by MTRFR deficiency, we needed to use a different approach for metabolic labeling in cultured cells. So far, our analysis reflected a steady equilibrium from the culmination of events that arise with MTRFR deficiency (Fig. 8, B to D, and fig. S4). To circumvent this, our goal was to reset mitochondrial protein synthesis and then monitor the incorporation of ^35^S-methionine/cysteine into individual polypeptide chains. First, we treated fibroblasts with puromycin to terminate protein synthesis followed by a 24-hour incubation with chloramphenicol to inhibit translation elongation on mitochondrial ribosomes (Fig. 8E). Chloramphenicol was then washed out followed by incubation with ^35^S-methionine/cysteine in fresh labeling media with anisomycin to inhibit cytosolic protein synthesis for a series of extended time points (Fig. 8E and fig. S5). This experimental manipulation revealed a differential effect on the synthesis of mitochondrial proteins with MTRFR deficiency (Fig. 8E). Several proteins exhibited a profound impairment in synthesis that did not correlate with known synthesis rates in fibroblasts or myoblasts (*41*) nor composition within an oxidative phosphorylation complex (Fig. 8E). For example, MT-ATP6, which encodes a stop codon within the mRNA (Fig. 1B and table S1) and is one of the most highly synthesized proteins (*41*), was profoundly inhibited by MTRFR deficiency. In contrast, the synthesis of MT-ND1, which does not encode a stop codon within the mRNA (Fig. 1B and table S1) and considered a low-expressed protein by ribosome profiling (*41*), was only modestly affected. The reduced synthesis was not due to changes in mitochondrial mRNA abundance. MTRFR deficiency shows no effect on the steady abundance of mitochondrial mRNAs (*33*), and our experimental manipulation to reset mitochondrial protein synthesis does not reduce transcript levels (Fig. 5B). It is also not due to an imbalance in protein synthesis between cellular compartments as the synthesis of the nuclear-encoded subunits of the oxidative phosphorylation complexes appears to be uncoupled from mitochondrially synthesized proteins in cultured human cells (*41*). Our data suggest that with MTRFR deficiency, there is a rapid impairment in mitochondrial protein synthesis for selective proteins, revealing the existence of far more complex gene expression regulation.

**Fig. 9.**
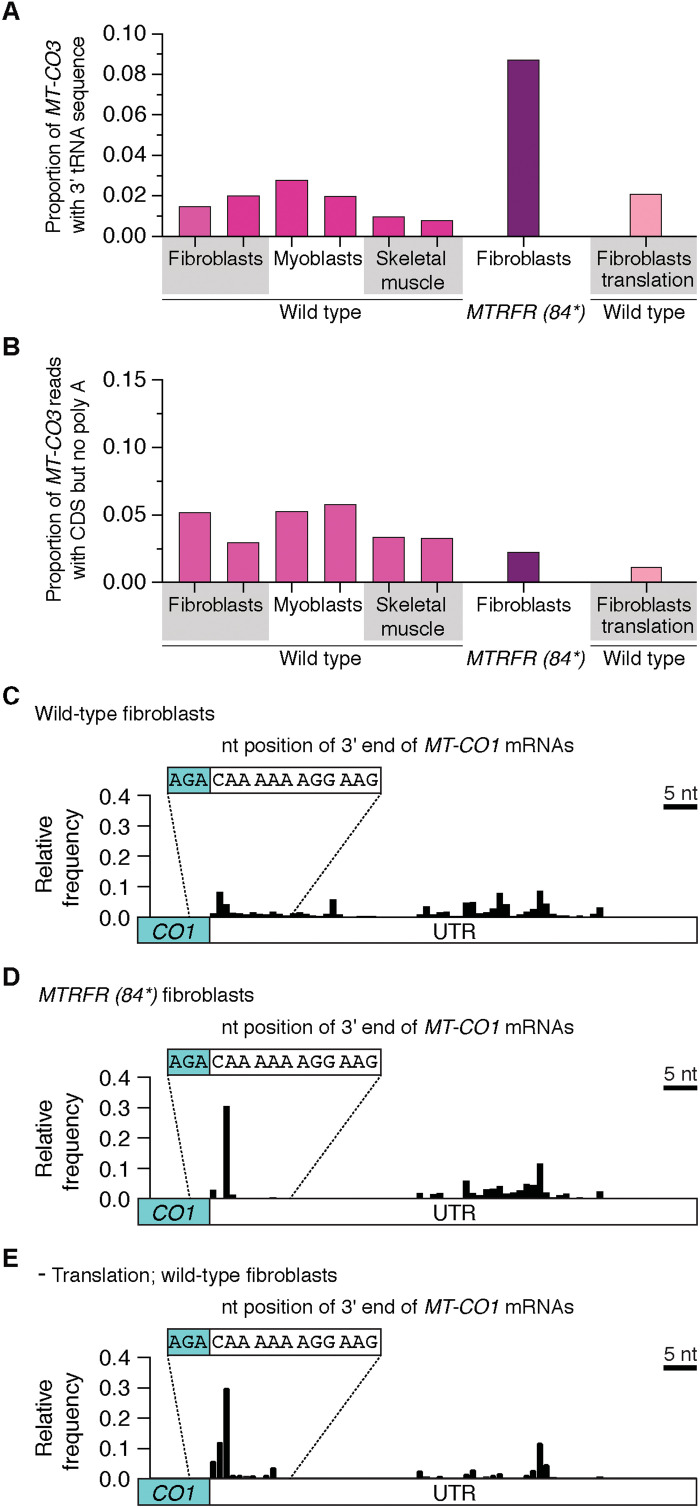
Mitochondrial nonstop mRNAs with MTRFR deficiency. (**A**) Deep sequencing of the 3′ end of *MT-CO3* mRNA from the whole transcriptome of biologically independent healthy controls taken from cultured fibroblasts, myoblasts, and human skeletal muscle biopsies compared to patient fibroblasts with MTRFR deficiency and where mitochondrial translation was inhibited with 24 hours of chloramphenicol treatment. Data show proportion of reads with CDS + TRNG at the 3′ end. (**B**) Proportion of sequencing reads where the 3′ end of the *MT-CO3* CDS is correctly processed but missing polyadenylation. (**C** to **E**) Frequency histogram indicating the mapped 3′ nt position from deep sequencing of *MT-CO1* mRNAs that were not polyadenylated isolated from the whole transcriptome of healthy control human cultured fibroblasts, MTRFR deficiency, and chloramphenicol inhibition of mitochondrial translation. Data analysis taken from 101,294 sequencing reads for wild-type fibroblasts, 308,099 for MTRFR deficiency, and 9113 for the translation inhibition.

A consequence from MTRFR deficiency could be the accumulation of nonstop mRNAs from the failure to terminate protein synthesis. Although we do not observe an increase in mitochondrial ribosome complexes with the sucrose gradient separation (Fig. 8D), the inability to effectively coordinate ribosome recycling could alter the abundance of these transcripts. To test this hypothesis, we turned to our deep sequencing approach in patient-derived fibroblasts with MTRFR deficiency. We focused on the *MT-CO1* and *MT-CO3* transcripts, which encode subunits of cytochrome c oxidase but lack a stop codon (Fig. 1C and table S1). The synthesis rates for both subunits is similar (*41*). *MT-CO3* is one of the six transcripts with a 3′ flanking tRNA that require RNA processing followed by polyadenylation to generate a stop codon (Figs. 1C and 4A and table S1). MTRFR-deficient cells exhibit a robust increase in the proportion of mRNA reads with aberrant 3′ ends that contain tRNA^Gly^ sequence (Fig. 9A), whereas unexpectedly, we detected no difference in the abundance of mRNAs that were correctly processed but lacking the polyadenylation modification to generate a stop codon (Fig. 9B). Further, inhibition of mitochondrial protein synthesis with chloramphenicol (Fig. 5) does not increase the abundance of *MT-CO3* nonstop mRNAs (Fig. 9, A and B). This would suggest that impaired mitochondrial protein synthesis per se does not account for the specific increase in *MT-CO3* nonstop mRNAs that we observed with MTRFR deficiency (Fig. 9A).

Next, we turned to the *MT-CO1* transcript, which encodes a long 3′ untranslated region (3′UTR) sequence following the terminal AGA codon (Figs. 1B and 9C and table S1) (*4*, *8*). Thus, for MTRFR to terminate translation in an analogous mechanism to ArfB would require truncations of the UTR no longer than the length of the mRNA channel from the A site to the entry site on the mitochondrial ribosome [approximately 36 Å or 12 nt (*42*)]. To test for this, we investigated the 3′ end of *MT-CO1* in wild-type fibroblasts. In the sequencing *MT-CO1* reads that lacked polyadenylation, we observed a subset with UTRs less than 9 nt (Fig. 9C). We observed a similar pattern in myoblasts and skeletal muscle (fig. S6). Although we analyzed the entire mitochondrial transcriptome, the data reveal MT-CO1 mRNAs with truncated 3′ ends that could be nonstop termination substrates. Unexpectedly, we observed a robust increase in truncated *MT-CO1* transcripts with MTRFR deficiency (Fig. 9D). However, this increase was also seen when mitochondrial protein synthesis is inhibited with chloramphenicol (Fig. 9E). The basis by which these truncated transcripts are generated is an open question; the data nonetheless reveal a translation-dependent mechanism for *MT-CO1* mRNA quality control.

To investigate the mechanism further, we analyzed that the 3′ end of *MT-ND6*, which encodes the AGG codon, has a long 3′UTR and is only mRNA on the light strand of the genome (Fig. 1, A and B, and fig. S7). The deep sequencing revealed a subset of *MT-ND6* mRNAs with truncated UTRs (fig. S7) in wild-type fibroblasts, myoblasts, and skeletal muscle. Notably, the level of these truncated transcripts was not altered with MTRFR deficiency nor with inhibition of mitochondrial protein synthesis (fig. S7). Together, the analysis reveals a subset of mRNAs whereby AGA or AGG could act as nonstop codons in mitochondrial gene expression and require MTRFR for termination according to our proposed model.

## DISCUSSION

Together, our collective data reveal sources by which nonstop mRNAs arise in mitochondrial gene expression and can act as a negative regulator of protein synthesis. In turn, this underpins a selective pressure to maintain the framework of a bacterial ribosome rescue pathway that is mediated by MTRFR. Our working model posits that MTRFR is recruited to mitochondrial ribosomes translating nonstop mRNAs. We favor an interpretation whereby the C-terminal α helix inserts into the mRNA channel of the mitochondrial ribosome to reposition the catalytic domain to the PTC in analogous mechanism to that of ArfB on bacterial ribosomes. The overwhelming structural conservation between the bacterial and human release factors and the functional importance of the MTRFR C terminus in mitochondrial protein synthesis are consistent with such an interpretation.

Across all domains of life, quality control mechanisms have evolved to recognize aberrations in protein synthesis. In bacteria, there is an intrinsically high rate of protein synthesis errors, but these are typically masked by nonstop rescue mechanisms (*43*). Three pathways have evolved to resolve these errors: trans-translation (transfer-messenger RNA), ArfA, and ArfB (*1*). All three systems are functionally unique for resolving protein synthesis termination on truncated transcripts, revealing a robust redundancy in ribosome quality control systems in bacteria (*44*). In contrast, cytoplasmic ribosomes have a distinct set of ribosome quality control factors activated in response to slow or stalled ribosomes that can arise from a plethora of causes, including mRNA defects (*2*). Considering the simplicity of human mitochondrial gene expression, it is perhaps not unexpected that only one pathway of proteobacterial origin would be retained, and ArfB is the simplest of those systems.

Because MTRFR dysfunction presents as a pleiotropic clinical spectrum with tissue-specific phenotypes (*45*, *46*), this suggests that nonstop ribosome complexes arise frequently in mitochondrial gene expression, vary across cell types, and contribute to molecular pathogenesis of mitochondrial disorders. At the clinical level, there is also a genotype-phenotype correlation so that, generally, the most severe presentations arise in patients when the C-terminal α helix is missing (*45*, *46*). However, there is one notable exception, whereby a patient with a functionally null pathogenic variant presented in the clinic in their mid-40s (*47*), which is at odds with the typical childhood onset. This finding could be indicative of genetic modifiers for MTRFR deficiency and/or mitochondrial ribosome quality control pathways.

In the case of the six human mitochondrial mRNAs that do not encode a stop codon within the CDS (Fig. 1B), translation of correctly processed transcripts but missing polyadenylation nonetheless generates fully functional mature polypeptides that are viable substrates in the de novo assembly of oxidative phosphorylation complexes. In contrast, translation of nonstop mRNAs that are truncated polyadenylated transcripts or have a flanking tRNA sequence will require termination followed by rapid proteolysis because of the inherent propensity of these aberrant nascent polypeptide chains to misfold. Defects in the strict cotranslational quality control of mitochondrial protein synthesis can exert rapid deleterious effects on mitochondrial membrane integrity, morphology, and organelle function as part of a feedback response onto gene expression regulation (*10*, *34*, *48*–*50*). These processes need to be considered when developing an integrated molecular understanding by which the defect in mitochondrial protein synthesis arises with MTRFR deficiency. Despite 12 of the 13 mitochondrial mRNAs transcribed in equal stoichiometry, the translation of different mRNAs by mitochondrial ribosomes differs significantly (*41*). The regulatory basis for this selectivity is currently not known. In the case of MTRFR deficiency, this mRNA selectivity, the translation frequency of nonstop mRNAs, and the potential for additional cotranslational regulatory events will likely determine how a general defect in mitochondrial protein synthesis arises. Together, data in this study provide further evidence for the inherent complexity of mitochondrial gene expression regulation and the need to elucidate the underlying molecular mechanisms to understand the pathogenesis of mitochondrial protein synthesis disorders (*3*).

The role of mL62 in this mitochondrial protein synthesis termination mechanism remains an open question. Although mL62 retains the conserved catalytic domain of a release factor, at the steady-state level in the cell, it is a structural component of the human mitochondrial ribosome and located too distal from the PTC to be functional in protein synthesis termination (*38*, *39*). That said, the recombinant protein can interact with mitochondrial ribosomes programmed on a truncated transcript in a reconstituted in vitro system (*25*, *28*, *51*). In cells, so far, the function of mL62 has been investigated in cultured proliferating adherent cell types (*52*). There could be compensatory responses of mL62 expression whereby the release factor does not assemble into the large subunit in a cell-specific manner. Studies investigating this hypothesis in primary cell types and tissues would be more definitive models for exploring these mechanisms.

The negative regulatory effect arising from translation of aberrant mRNAs may hold further relevance to the molecular pathogenesis of other mitochondrial disorders. Because of the multicopy nature of the mitochondrial genome, most pathogenic heteroplasmic mtDNA variants are functionally recessive (*53*). An exception to this are large-scale rearrangements or deletions of the circular mitochondrial genome, which tend to exert a functionally dominant-negative effect on the synthesis of wild-type subunits of the oxidative phosphorylation complexes (*54*). The molecular basis of this mechanism has been largely unexplored. We propose the following model to reconcile the previous observations with our current analysis in this study. Because transcription of genomes with deletions will generate fusion ORFs (*55*), the apparent absence of strict RNA surveillance mechanisms to prevent translation of mRNAs with aberrant 3′ ends will compound the error rate in protein synthesis. This could, in turn, sequester the associated ribosome quality control factors away from resolving the baseline translation errors arising from nonstop mRNAs, leading to the accumulation of mitochondrial ribosomes that cannot be recycled. Impaired ribosome recycling would disrupt protein synthesis and prevent the synthesis of wild-type oxidative phosphorylation subunits even with a high wild-type mtDNA copy number in the cell. Moreover, such a mechanism could account for the focal accumulation of mtDNA deletions in the skeletal muscle (*54*). Thus, the inherent ability of a given cell type to generate, recognize, and resolve errors in protein synthesis may be a key factor in the molecular pathogenesis of mtDNA deletions. Further studies need to start exploring these regulatory mechanisms in primary cell types and tissues affected in human mitochondrial protein synthesis disorders.

## MATERIALS AND METHODS

### Biological samples

Human fibroblasts were cultured at 37°C and 5% CO_2_ in Dulbecco’s modified Eagle’s medium (Sigma-Aldrich) with high glucose supplemented with 10% fetal bovine serum, 1× glutamax, and uridine (50 μg/ml). Fibroblasts with segregating biallelic pathogenic variants (p.Arg445Gln and p.Ser400IlefsX6) in *PRORP* (NM_014672.3) exhibit a mitochondrial tRNA processing defect and organelle dysfunction similarly to other reported variants (*15*). C12orf65 (MTRFR)–deficient fibroblasts with stop-gain mutation at amino acid 84 have been previously reported (*33*, *34*). Two biologically independent human myoblast cultures were grown in myoblast medium (Sigma-Aldrich) supplemented with uridine (50 μg/ml). All cells tested negative for mycoplasma infection (PromoKine). Two biologically independent human skeletal muscle biopsies were acquired from healthy controls, unrelated to the myoblast cultures, with prior informed consent during routine anterior cruciate ligament surgery following ethical approval granted by Newcastle and North Tyneside Research Ethics Committees (REC: 12/NE/0394).

### Isokinetic sucrose gradients

Following procedures previously described (*10*), cells were cultured on 150-mm plates and then rapidly transferred to ice where the medium was removed, and cells were washed with cold phosphate-buffered saline. Cells were lysed [50 mM tris (pH 7.2), 10 mM Mg(Ac)_2_, 40 mM NH_4_Cl, 100 mM KCl, 1% dodecyl-maltoside (DDM), 1 mM adenosine triphosphate (ATP), chloramphenicol (400 μg/ml), and 1 mM phenylmethylsulfonyl fluoride (PMSF)] and incubated on ice for 20 min. Cell lysates were clarified following centrifugation for 10 min at 20,000*g* at 4°C, and then protein concentrations were measured (Bradford). From each cell lysate, a total of 1 mg of protein was loaded on top of a 16-ml linear 10 to 30% sucrose gradient [50 mM tris (pH 7.2), 10 mM Mg(Ac)_2_, 40 mM NH_4_Cl, 100 mM KCl, 1 mM ATP, and 1 mM PMSF] and centrifuged for 15 hours at 4°C and 74,400*g* (Beckman SW 32.1 Ti). From the gradient, 24 equal volume fractions were collected for either protein or RNA isolation. Samples for protein analysis were precipitated with trichloroacetic acid (*34*). For RNA isolation, fractions were combined according to the established sedimentation profile of mitochondrial ribosomes and then concentrated using a Microsep centrifugal filter with a molecular weight cutoff (MWCO) of 10 kDa at 7500*g* for 90 min at 4°C. RNA was isolated using TRIzol LS reagent (Thermo Fisher Scientific).

### RNA sequencing library preparation and analysis comparing the total mitochondrial transcriptome to that associated with mitochondrial ribosomes

RNA was isolated with the Monarch Total RNA Miniprep Kit New England Biolabs (NEB), and 2 μg was fragmented using the NEBNext Magnesium RNA Fragmentation Module (94°C for 4 min) (200- to 300-nt size range) and then treated with T4 polynucleotide kinase (NEB) for 30 min to remove 3′ phosphates. Subsequently, RNA was cleaned up by using the Zymo RNA Clean & Concentrator-5 columns. The fragment size range and RNA concentration were verified on an Agilent Bioanalyzer with the RNA nano chip. Libraries were prepared using the template switching TGIRT (InGex) reverse transcriptase for cDNA synthesis (*9*, *56*) with three independent replicates prepared for each sample. Briefly, pre-annealing of the R2R DNA/R2 RNA primer mixture was set up in a 30-μl reaction containing 10× reaction buffer [100 mM tris-HCl (pH 7.5) and 10 mM EDTA], R2R DNA (1 μM), and R2 RNA (1 μM), followed by an incubation at 85°C for 2 min and then cooled to 25°C with 3% ramp. For template switching reverse transcription, 2 μl of the pre-annealed primer mixture, 5× reaction buffer [2.25 M NaCl, 25 mM MgCl_2_, and 100 mM tris-HCl (pH 7.5)], 10× dithiothreitol (50 mM), 50 ng of RNA, and TGIRT-III (InGex) were combined and preincubated at room temperature for 30 min, followed by the addition of 2 μl of 10 mM deoxynucleotide triphosphates. The reaction mixture was incubated at 60°C for 1 hour and terminated with 1 μl of 5 M NaOH, followed by heating at 95°C for 3 min and then neutralized with 1 μl of 5 M HCl. The cDNAs were cleaned up via the MinElute Reaction Cleanup Kit (Qiagen) and repeated twice to decrease the amount of unused R2R DNA adapters. The R1R DNA adapter was pre-adenylated using the 5′ DNA Adenylation Kit (NEB) and then ligated to the 3′ end of the cDNA using the thermostable 5′ App DNA/RNA Ligase (NEB) for 2 hours at 65°C. Ligated products were purified twice using the MinElute columns. A quarter of the eluted cDNA ligation products were amplified by polymerase chain reaction (PCR) with Phusion High-Fidelity DNA polymerase (Thermo Fisher Scientific; 21 cycles). PCR products were cleaned up with magnetic beads (MagSi NGSPREP Plus) and sequenced on an Illumina NextSeq 500 for 75–base pair (bp) paired-end reads (a total of 67 and 73 million reads per sample).

Illumina sequencing reads were analyzed by FastQC (v0.11.8) (*57*) (www.bioinformatics.babraham.ac.uk/projects/fastqc/). The paired and unpaired reads were separated using repair.sh function from BBQ (v38.79; https://sourceforge.net/projects/bbmap/). The adapters [for 3′ end sequencing including one extra nucleotide due to the variability at 5′ position (A, G, T, and C) of the R2R primer], barcodes, low-quality reads, and low-quality bases are removed using bbduk.sh from the same bbmap tool (v38.79) (trimq = 20, qin = auto, minlen = 10, qout = auto, qtrim = r and qtrim = rl). High-quality paired-end reads were aligned to *Homo sapiens* mitochondrion genome (accession number J01415.2) using bwa (v0.7.17) (*58*), and the coverage was calculated using genomecov from bedtools. The Kallisto quant (v0.44.0; pair-end, -b: 4000, --plaintext) was used as the pseudo-aligners, and the resulting raw count table was transferred to R environment by tximport package (v1.18.0; gene level expression and default mode). Differential expression analysis was carried out using EdgeR (v3.32.1; normalization method: Trimmed Mean of M-values (TMM); false discovery rate ≤ 0.05) comparing the monosome versus total RNA (*n* = 3 for each group).

### RNA sequencing library preparation and analysis for the 3′ end of mitochondrial mRNAs

RNA was isolated with the Monarch Total RNA Miniprep Kit (NEB) or the Reliaprep RNA Miniprep system (Promega) followed by deoxyribonuclease I (NEB) treatment. For each sample, 1 μg of RNA was treated with T4 polynucleotide kinase (NEB) to remove the 3′ phosphate and then purified using Agencourt AMPure XP magnetic beads (Beckman Coulter). Template switching reverse transcription cDNA synthesis with TGIRT was performed as described previously and cleaned up using MinElute columns. In a 25-μl reaction, 10 μl of cDNAs was PCR-amplified with Phusion High-Fidelity, 1.2 μl of gene-specific primers (table S1) mixture (5.6 μM each primer), and 1 μl of 10 μM R2R primer (UR166) at 98°C for 90 s, followed by 12 cycles of 98°C for 10 s, 64°C for 27 s, and 72°C for 27 s, final extension at 72°C for 7 min, and held at 4°C. PCR products were bar-coded for sequencing on one of two Illumina platforms. For the cultured human fibroblast samples, three independent libraries were generated and sequenced on a MiSEQ with 300-bp paired-end reads (a total of 1 million to 2.4 million reads per sample). Three independent libraries were generated for the cultured human myoblasts and two for the skeletal muscle biopsies and sequenced on a NextSeq 500 for 150-bp paired-end reads (a total of 5.1 million to 7.9 million reads per sample).

Deep sequencing Illumina reads of the mitochondrial 3′ end were analyzed with FastQC (v0.11.8) (*57*) (www.bioinformatics.babraham.ac.uk/projects/fastqc/). Paired and unpaired reads were separated using repair.sh function from BBQ (v38.79; https://sourceforge.net/projects/bbmap/). Adapters [for 3′ end sequencing including one extra nucleotide due to the variability at 5′ position (A, G, T, and C) of the R2R primer], barcodes, low-quality reads, and low-quality bases are removed using bbduk.sh from the same bbmap tool (v38.79) (trimq = 20, qin = auto, minlen = 10, qout = auto, qtrim = r and qtrim = rl). High-quality sequencing reads were then aligned to the *H. sapiens* reference mitochondrial genome, using BLAST (v2.6.0; max_target_seqs: 1) (*59*), and subsequently sorted by custom R codes. Reads with an exact match to the 3′ CDS of each mRNA were classified as having the true 3′ end of the CDS and then further sorted into distinct bins based on the potential intervening sequence up to 5′ of the R2R primer. These include no sequence extension (CDS), oligo and polyadenylation (CDS + poly A), and flanking UTR (CDS + UTR). Sequencing reads containing at least +3 nt of a flanking tRNA sequence were classified as CDS + tRNA. Truncated reads missing −3 nt from the 3′ nt of the CDS and having posttranscriptional adenylation were further retained for analysis. Sequencing reads generated from each independent RNA sequencing library were preprocessed and analyzed individually with summary figures presenting the pooled results of the replicates for each sample.

### Northern blotting

Total cellular RNA was isolated using the Reliaprep RNA Miniprep system (Promega) according to the manufacturer’s instructions. For all samples, 5 μg of total RNA was run through 1.2% agarose-formaldehyde gels and transferred to Hybond-N+ membrane (GE Healthcare) by neutral transfer. T4 polynucleotide kinase (NEB) 5′-radiolabeled oligonucleotides were used for detection of mitochondrial transcripts. Oligonucleotides are as follows: 16*S*, 5′-GCTGTGTTATGCCCGCCTCTTCACGGG; MT-ATP8/6, 5′-TGGGTGATGAGGAATAGTGTAAGGAG; MT-ND4, 5′-AGTCAGGTAGTTAGTATTAGGAGGG; MT-CYTB, 5′-GCGGTTGAGGCGTCTGGTGAGTAGTGC; MT-CO1, 5′-GGCTCCAGGGTGGGAGTAGTTCCCTGC. Hybridization [25% formamide, 7% SDS, 1% bovine serum albumin (BSA), 0.25 M sodium phosphate (pH 7.2), 1 mM EDTA (pH 8.0), and 0.25 M NaCl_2_] was performed for 16 to 20 hours at 37°C. Membranes were washed (2× SSC/0.1% SDS), then dried for exposure on a phosphoscreen (GE Healthcare), and scanned with a Typhoon 9400 (GE Healthcare).

### In vitro translation

The PURExpress ΔRF123 cell-free translation system (NEB) was used with the following modifications. DNA templates were generated by PCR using the supplied dihydrofolate reductase (DHFR) plasmid as a template with the respective primers (table S1). Each construct had an upstream T7 promoter sequence and a Shine-Dalgarno sequence of the DHFR ORF, according to the manufacturer’s instructions. Equimolar reactions of each specific construct were prepared for in vitro transcription and translation reactions in a total volume of 12.5 μl containing 5 μl of PURExpress kit solution A, 3.75 μl of solution B, 0.25 μl each of the supplied release factor (RF1, RF2, and RF3), 8 U of RiboLock RNase inhibitor (Thermo Fisher Scientific), 3 ng of DNA template, 5 μM anti-ssrA oligonucleotide (*60*), and 500 μCi of ^35^S Met-Cys (EasyTag-PerkinElmer) and incubated at 37°C. An equal volume of gel loading buffer [186 mM tris-HCl (pH 6.7), 15% glycerol, 2% SDS, and bromophenol blue (0.5 mg/ml)] was added to the samples and incubated at room temperature for 60 min. Translation products were resolved on 10% NuPAGE bis-tris gels (Thermo Fisher Scientific) in Mops running buffer (50 mM Mops, 50 mM tris, 1 mM EDTA, and 0.1% SDS). In the competition experiments, reactions were set up with equimolar amounts of *DHFR* stop and *ND3* nonstop templates. All samples were treated with Benzonase (Sigma-Aldrich) on ice for 30 min to hydrolyze any polypeptidyl-tRNAs before the addition of the gel loading buffer. Gels were dried under vacuum, then exposed with a phosphorimager screen, and scanned with Typhoon 9400 (GE Healthcare) for quantification.

### Cloning and retroviral transduction of patient-derived MTRFR-deficient fibroblasts

The full-length wild-type cDNA of human *C12orf65* and the catalytic mutant GSQ were previously cloned into the retroviral expression vector pBABE-puro (*34*). The wild-type cDNA was used as a template to generate the C-terminal deletion series (e.g., 141, 151, and 160) using PCR followed by Gateway cloning into pBABE-puro. A wild-type *ICT1* cDNA clone was obtained from the ORFeome collection (clone no. 100003285) followed by Gateway cloning into pBABE-puro. All constructs were verified by Sanger sequencing. Retrovirus was generated by transient transfection of retroviral plasmids into the Phoenix amphotropic packaging line as previously described (*34*). Virus was collected and then used to infect recipient cells, which were used directly in experiments following selection with puromycin. Data presented are representative of multiple independent retroviral experiments. For deep sequencing of the mitochondrial 3′ ends with MTRFR deficiency, total RNA was isolated from cultured patient-derived fibroblasts stably transduced with the catalytically dead GSQ mutant with libraries prepared for deep sequencing as described above.

### Immunoblotting

Lysates were prepared in phosphate-buffered saline, 1% DDM, 1 mM PMSF (Sigma-Aldrich), and complete protease inhibitor (Thermo Fisher Scientific). Protein concentrations were measured by the Bradford assay (Bio-Rad), and equal amounts were separated by 12% tris-glycine SDS–polyacrylamide gel electrophoresis (SDS-PAGE) and transferred to nitrocellulose membrane by semidry transfer. Membranes were blocked in TRIS-buffered saline with 2% Tween 20 (TBST) with 1% milk at room temperature for 1 hour, followed by incubation with primary antibodies overnight at +4°C in 5% BSA/TBST, and detected the following day with secondary horseradish peroxidase conjugates (Jackson ImmunoResearch) using LumiGLO ECL (Cell Signaling Technology) with film. Primary antibodies from Proteintech Group are as follows: uL11m (1:20,000; 15543-1-AP), mL45 (1:10,000; 15682-1-AP), mS27 (1:5000; 17280-1-AP), mS35 (1:5000; 16457-1-AP), MT-ATP6 (1:2000; 55313-1-AP), and LRPPRC (1:8000; 21175-1-AP). The primary antibody from Santa Cruz Biotechnology is TOM40 (1:2000; sc-11414). Primary antibodies from MitoSciences/Abcam are as follows: MT-CO1 (1:500; 1D6E1A8) and SDHA (1:10,000; ab-14715). Primary antibody from Sigma-Aldrich is MTRES1 (1:1000; HPA049535). Representative data of independent experiments were cropped in Adobe Photoshop with only linear corrections to brightness applied.

### Radioisotope labeling of mitochondrial translation

A 30-min pulse labeling of mitochondrial nascent chains with ^35^S Met-Cys (EasyTag-PerkinElmer) in cultured cells was performed as described (*48*). All samples were treated with Benzonase according to the manufacturer’s instructions and then mixed with gel loading buffer [186 mM tris-HCl (pH 6.7), 15% glycerol, 2% SDS, bromophenol blue (0.5 mg/ml), and 6% β-mercaptoethanol]. A 12 to 20% gradient SDS-PAGE was used to separate samples, then dried for exposure with a Phosphoscreen, and scanned with a Typhoon 9400 (GE Healthcare) for quantification. Gels were rehydrated in water and Coomassie-stained to confirm loading.

### Phylogenetic analysis of MT-CO1 and MT-ND6

Mammalian mtDNA RefSeq accessions (RefSeq release 202) were obtained from the Organelle Genome Resources page of the National Center for Biotechnology Information (NCBI) (www.ncbi.nlm.nih.gov/genome/organelle/), and associated sequences were retrieved by use of the NCBI Entrez utilities (www.ncbi.nlm.nih.gov/books/NBK25501/). Duplicate sequences were removed by seqkit v0.13.2 (*61*), and the remaining sequences were aligned using the FFT-NS-2 method under MAFFT v7.471 (*62*). SequenceBouncer v1.13 (*63*) was used with a *k*-setting of 0.75 and a gap threshold of 2% to remove aberrant and outlier entries. Subsequently, the “taxize” package (*64*) and the NCBI taxonomy database (*65*) were used when reducing the alignment to haplorhine mtDNA sequences. Any sequence with an accession not starting with “NC_” was removed, and the resulting sequence set was realigned by MAFFT v7.471 using the FFT-NS-2 approach. Relevant regions of the *MT-COI* and *MT-ND6* genes were ungapped using seqkit v0.13.2 for downstream analyses.
