# Supplementary material for: Nonstop mRNAs generate a ground state of mitochondrial gene expression noise
Source: Sci Adv. 2022 Nov 18;8(46):eabq5234. doi: 10.1126/sciadv.abq5234 (PMC9674279; doi:10.1126/sciadv.abq5234)
Supplement: Supplementary file 1 — Figs. S1 to S7 Table S1 [file sciadv.abq5234_sm.pdf]

Science Advances
AAAS

Supplementary Materials for

Nonstop mRNAs generate a ground state of mitochondrial gene expression noise

Kah Ying Ng *et al.*

Corresponding author: Brendan J. Battersby, brendan.battersby@helsinki.fi

Sci. Adv. **8**, eabq5234 (2022)
DOI: 10.1126/sciadv.abq5234

This PDF file includes:

Figs. S1 to S7
Table S1

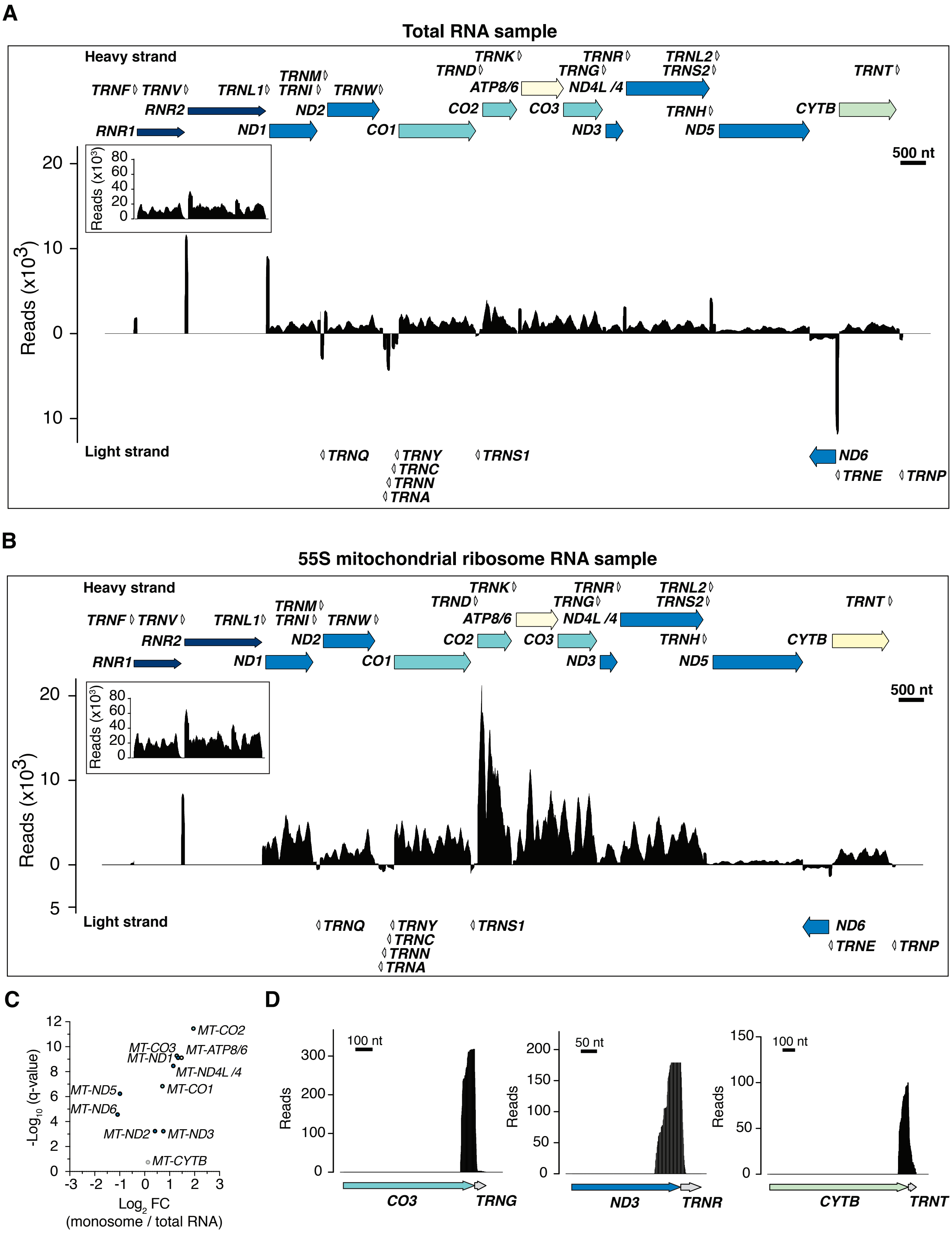
A
Total RNA sample
Heavy strand
TRNF TRNV TRNL1 TRNM TRNI TRNW TRNK TRND ATP8/6 TRNR TRNG ND4L /4 TRNL2 TRNS2 TRNT
RNR2 ND2 CO2 CO3 TRNH CYTB
RNR1 ND1 CO1 ND3 ND5
500 nt
Reads (x10^3)
20 10 0 10
80 60 40 20 0
Light strand
TRNQ TRNY TRNC TRNN TRNA TRNS1 ND6 TRNE TRNP
B
55S mitochondrial ribosome RNA sample
Heavy strand
TRNF TRNV TRNL1 TRNM TRNI TRNW TRNK TRND ATP8/6 TRNR TRNG ND4L /4 TRNL2 TRNS2 TRNT
RNR2 ND2 CO2 CO3 TRNH CYTB
RNR1 ND1 CO1 ND3 ND5
500 nt
Reads (x10^3)
20 10 0 5
80 60 40 20 0
Light strand
TRNQ TRNY TRNC TRNN TRNA TRNS1 ND6 TRNE TRNP
C
-Log$_{10}$ (q-value)
12 10 8 6 4 2 0
MT-CO2
MT-CO3
MT-ATP8/6
MT-ND1
MT-ND4L /4
MT-ND5
MT-CO1
MT-ND6
MT-ND2
MT-ND3
MT-CYTB
-3 -2 -1 0 1 2 3
Log$_2$ FC
(monosome / total RNA)
D
Reads
300 200 100 0
100 nt
CO3 TRNG
200 150 100 50 0
50 nt
ND3 TRNR
150 100 50 0
100 nt
CYTB TRNT

Fig. S1: Deep sequencing analysis of mitochondrial RNA using the group II intron reverse transcriptase (TGIRT) provides robust unbiased coverage of the transcriptome and reveals aberrant tRNA processing. (**A**), Mapped sequencing reads from RNA isolated from the whole cell RNA prepared from the HEK293 cell line. Inset box, mapping of rRNA. (**B**), Mapped sequencing reads from RNA isolated from mitochondrial ribosomes. In the human mitochondrial ribosome, the $tRNA^{Val}$ has been incorporated as a structural component and thus notably enriched in these preparations. (**C**) As a measure of translation efficiency, the fold change (FC) in mRNA abundance sequenced from the mitochondrial ribosome was normalized to the total mRNA abundance. (**D**), Examples of deep sequencing reads of mRNA (*MT-CO3, ND3* and *CYTB*) with the mapped 3' end containing flanking tRNA sequence. RNA isolated from mitochondrial ribosomes following sucrose density gradient separation.

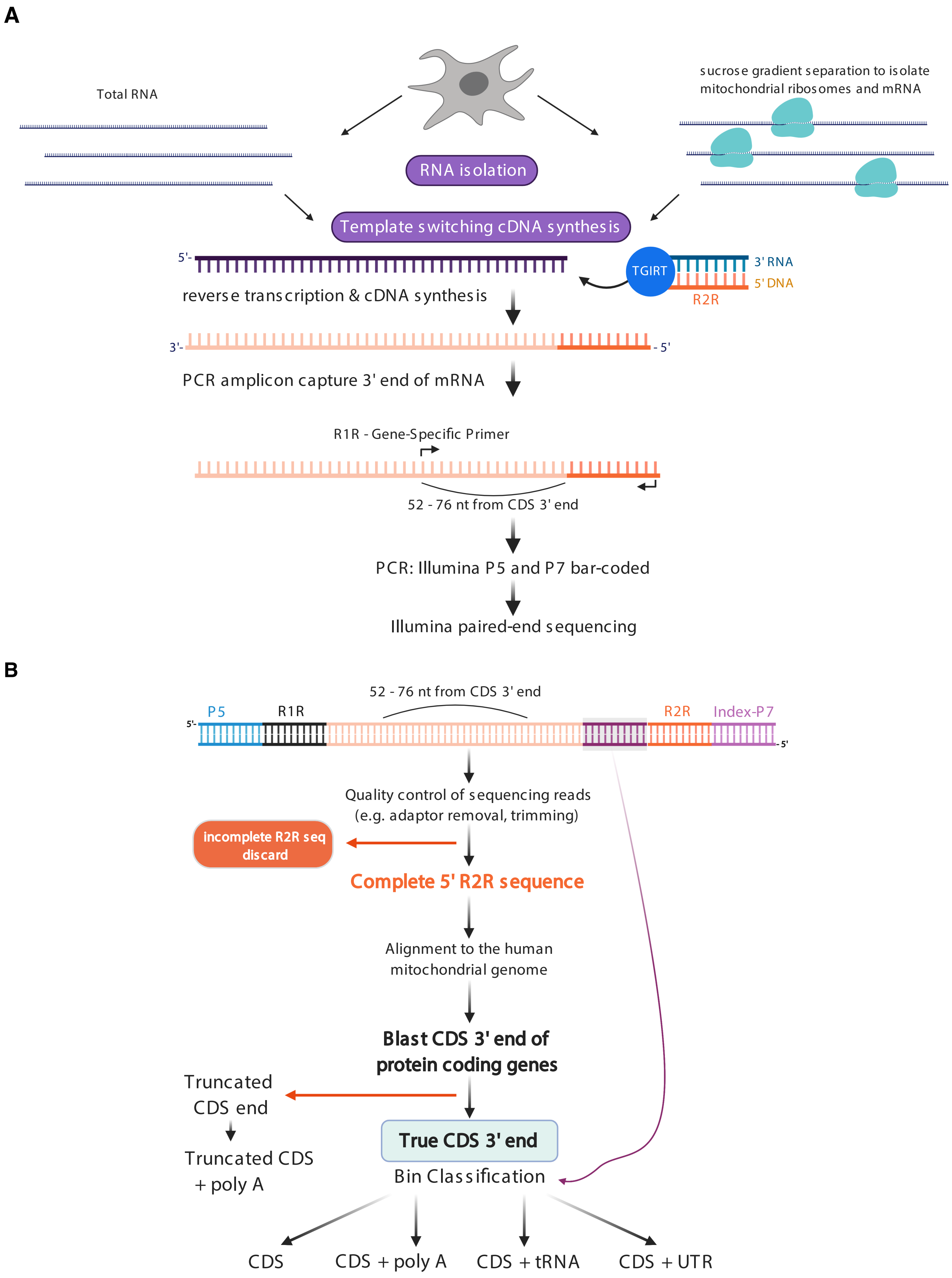
A
Total RNA
sucrose gradient separation to isolate mitochondrial ribosomes and mRNA
RNA isolation
Template switching cDNA synthesis
5'-
TGIRT
3' RNA
5' DNA
R2R
reverse transcription & cDNA synthesis
3'-
- 5'
PCR amplicon capture 3' end of mRNA
R1R - Gene-Specific Primer
52 - 76 nt from CDS 3' end
PCR: Illumina P5 and P7 bar-coded
Illumina paired-end sequencing
B
52 - 76 nt from CDS 3' end
P5
R1R
R2R
Index-P7
5'-
-5'
Quality control of sequencing reads (e.g. adaptor removal, trimming)
incomplete R2R seq discard
Complete 5' R2R sequence
Alignment to the human mitochondrial genome
Blast CDS 3' end of protein coding genes
Truncated CDS end
Truncated CDS + poly A
True CDS 3' end
Bin Classification
CDS
CDS + poly A
CDS + tRNA
CDS + UTR

Fig. S2: Deep sequencing approach to analyze specifically the 3' end of mitochondrial mRNA using the group II intron reverse transcriptase (TGIRT). (**A**) Schematic of the RNA sample isolation, cDNA synthesis, and library preparation for deep sequencing. (**B**) Workflow of the bioinformatic pipeline to analyze Illumina sequencing reads. Images created with BioRender.com.

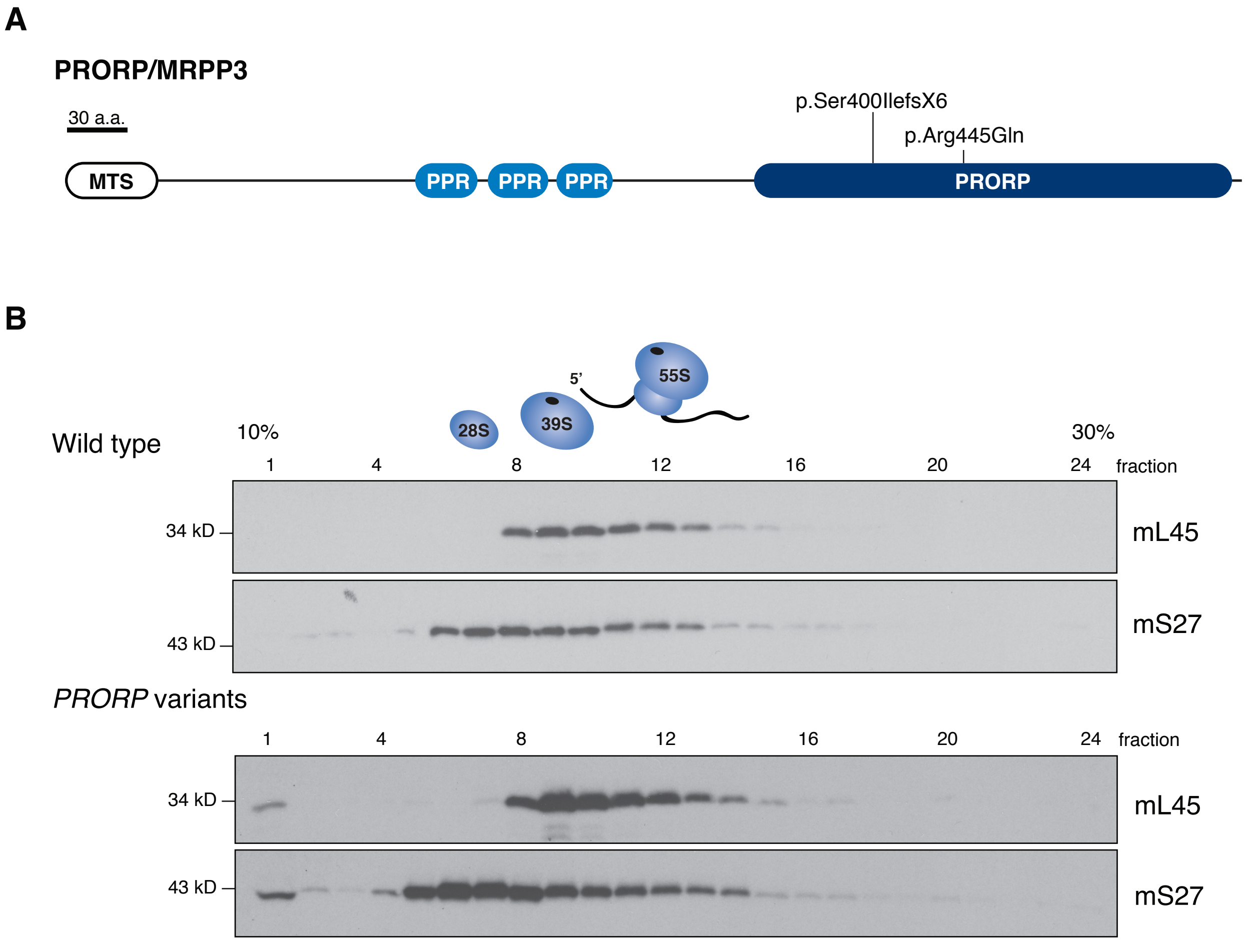
A
PRORP/MRPP3
30 a.a.
p.Ser400IlefsX6
p.Arg445Gln
MTS
PPR
PPR
PPR
PRORP
B
55S
5'
28S
39S
Wild type
10%
30%
1
4
8
12
16
20
24
fraction
34 kD
mL45
43 kD
mS27
PRORP variants
1
4
8
12
16
20
24
fraction
34 kD
mL45
43 kD
mS27

Fig. S3: Pathogenic variants in *PRORP* disrupt mitochondrial RNA processing but do not impair the assembly of mitochondrial ribosomes. (**A**) Schematic of the protein domains for PRORP and the location of pathogenic variants. MTS, mitochondrial targeting sequence. PPR, pentatricopeptide repeat. PRORP, protein-only RNase P. (**B**) Sucrose density gradient separation of mitochondrial ribosomes from cultured human fibroblasts with the indicated genotypes.

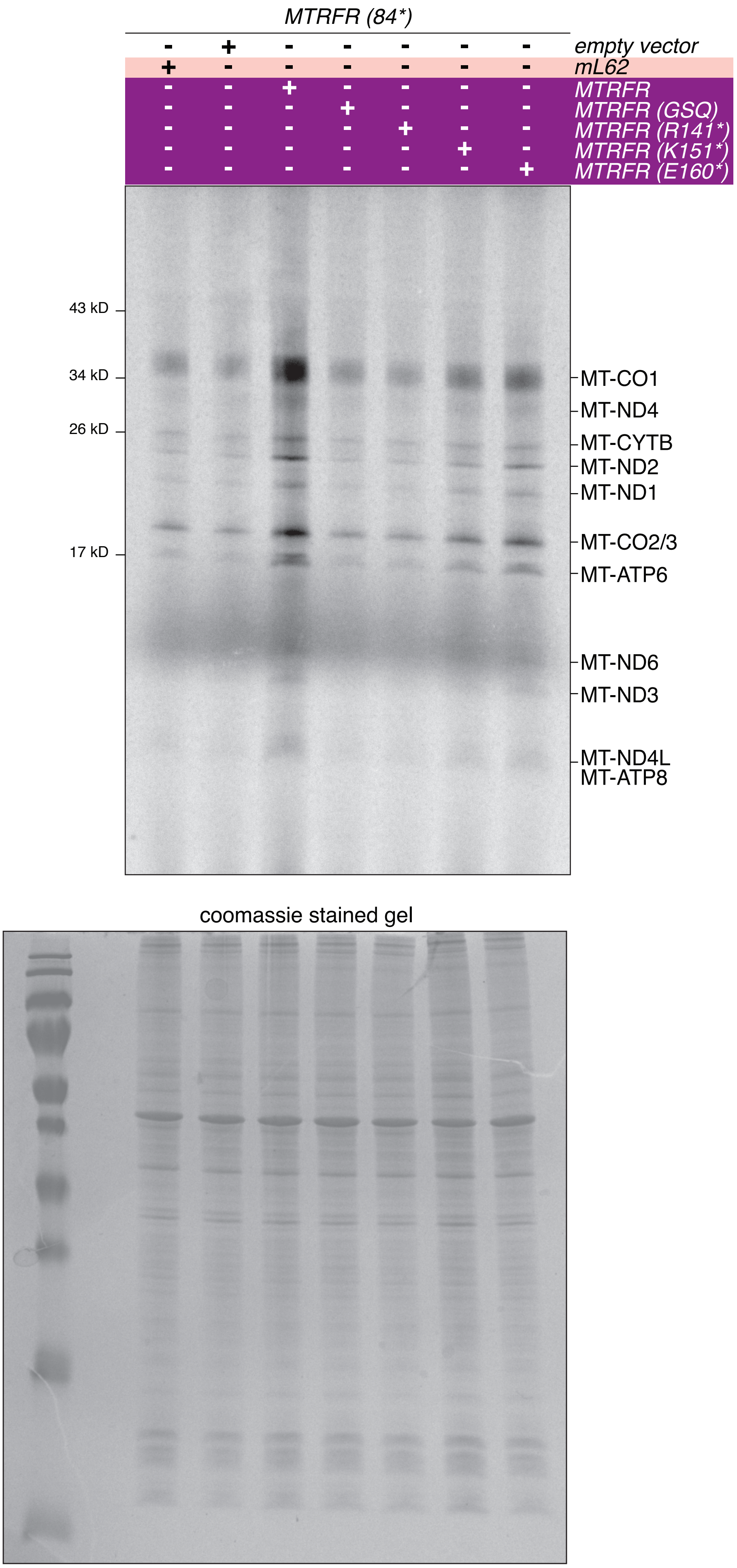
MTRFR (84*)
empty vector
mL62
MTRFR
MTRFR (GSQ)
MTRFR (R141*)
MTRFR (K151*)
MTRFR (E160*)
43 kD
34 kD
26 kD
17 kD
MT-CO1
MT-ND4
MT-CYTB
MT-ND2
MT-ND1
MT-CO2/3
MT-ATP6
MT-ND6
MT-ND3
MT-ND4L
MT-ATP8
coomassie stained gel

Fig. S4: Impaired mitochondrial protein synthesis with MTRFR deficiency. A representative ^{35}S- metabolic pulse labelling experiment in MTRFR-deficient (84*) fibroblasts stably transduced with cDNAs of the indicated MTRFR alleles and wild type *mL62.* Following exposure to a phosphoimaging screen the gel was hydrated and stained with coomassie to verify equal loading.

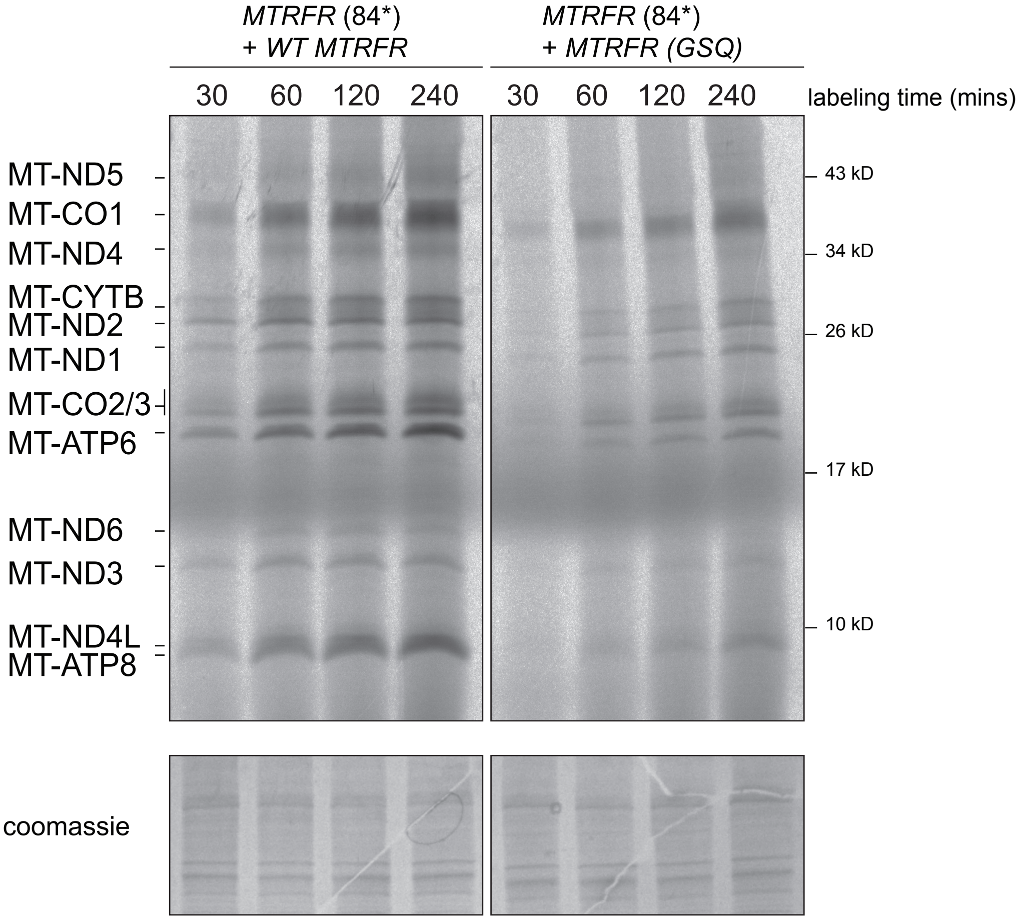

MTRFR (84*)
+ *WT MTRFR*
MTRFR (84*)
+ *MTRFR (GSQ)*
30
60
120
240
30
60
120
240
labeling time (mins)
MT-ND5
MT-CO1
MT-ND4
MT-CYTB
MT-ND2
MT-ND1
MT-CO2/3
MT-ATP6
MT-ND6
MT-ND3
MT-ND4L
MT-ATP8
43 kD
34 kD
26 kD
17 kD
10 kD
coomassie

Fig. S5: Impaired mitochondrial protein synthesis with MTRFR deficiency. A representative ^{35}S- metabolic extended pulse labeling experiment in MTRFR-deficient (84*) fibroblasts stably transduced with cDNAs of the indicated MTRFR alleles. Prior to labeling, cells were first treated with puromycin to terminate protein synthesis followed by a quick wash in PBS then a 24-hour incubation with chloramphenicol to inhibit mitochondrial translation elongation. Cells were washed in PBS followed by incubation with fresh labeling media (minus methionine and cysteine) in the presence of anisomycin for 5 minutes prior to adding ^{35}S-methionine/cysteine. Cell lysates were then separated by SDS-PAGE, dried, and exposed to a phosphoimaging screen. Subsequently, the gel was hydrated and stained with coomassie to verify equal loading. Data is representative of three independent experiments.

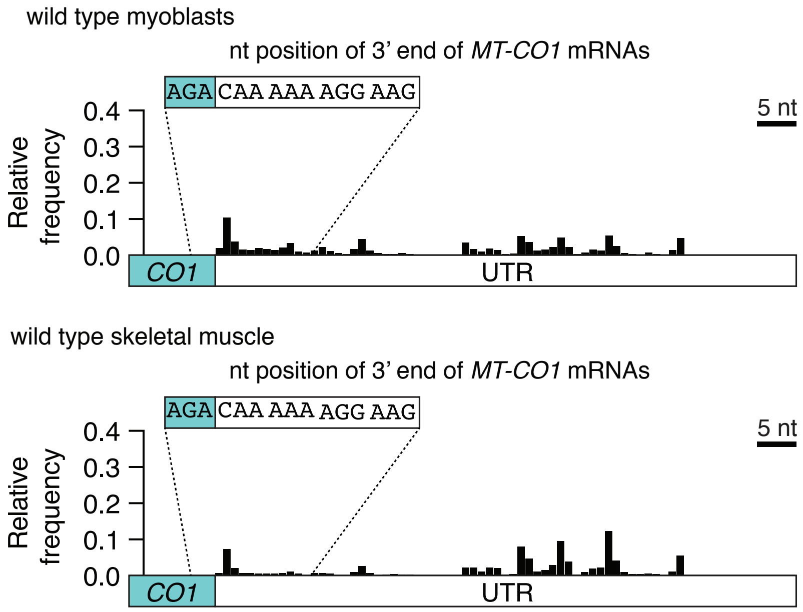
wild type myoblasts
nt position of 3' end of MT-CO1 mRNAs
AGA CAA AAA AGG AAG
5 nt
Relative frequency
0.4
0.3
0.2
0.1
0.0
CO1
UTR
wild type skeletal muscle
nt position of 3' end of MT-CO1 mRNAs
AGA CAA AAA AGG AAG
5 nt
Relative frequency
0.4
0.3
0.2
0.1
0.0
CO1
UTR

Fig. S6: Mapping the 3' nucleotide for *MT-CO1* mRNAs. Frequency histograms indicating the mapped 3' nucleotide position from deep sequencing of *MT-CO1* mRNAs that were not polyadenylated and isolated from the whole transcriptome of healthy wild type myoblasts and skeletal muscle. Data analysis taken from 47 818 sequencing reads for myoblasts and 176 107 for skeletal muscle.

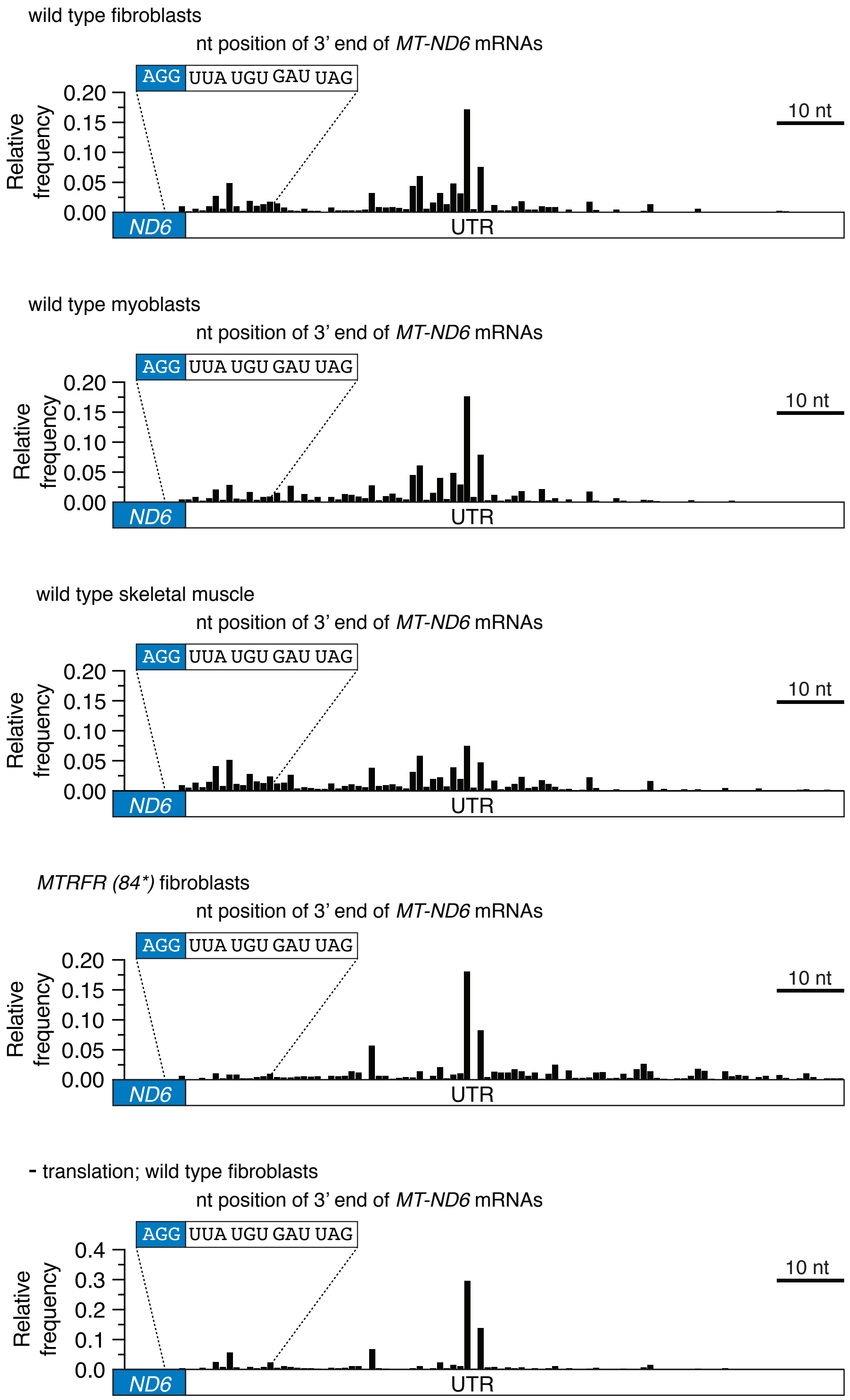
wild type fibroblasts
nt position of 3’ end of MT-ND6 mRNAs
AGG UUA UGU GAU UAG
Relative frequency
0.20
0.15
0.10
0.05
0.00
10 nt
ND6
UTR
wild type myoblasts
nt position of 3’ end of MT-ND6 mRNAs
AGG UUA UGU GAU UAG
Relative frequency
0.20
0.15
0.10
0.05
0.00
10 nt
ND6
UTR
wild type skeletal muscle
nt position of 3’ end of MT-ND6 mRNAs
AGG UUA UGU GAU UAG
Relative frequency
0.20
0.15
0.10
0.05
0.00
10 nt
ND6
UTR
MTRFR (84*) fibroblasts
nt position of 3’ end of MT-ND6 mRNAs
AGG UUA UGU GAU UAG
Relative frequency
0.20
0.15
0.10
0.05
0.00
10 nt
ND6
UTR
- translation; wild type fibroblasts
nt position of 3’ end of MT-ND6 mRNAs
AGG UUA UGU GAU UAG
Relative frequency
0.4
0.3
0.2
0.1
0.0
10 nt
ND6
UTR

Fig. S7: Mapping the 3' nucleotide for *MT-ND6* mRNAs. Frequency histograms indicating the mapped 3' nucleotide position from deep sequencing of *MT-ND6* mRNAs that were not polyadenylated and isolated from the whole transcriptome of healthy wild type fibroblasts, myoblasts, skeletal muscle, MTRFR deficiency, and chloramphenicol inhibition of mitochondrial translation. Data analysis taken from 59 638 sequencing reads for fibroblasts, 14 594 for myoblasts, 12 729 for skeletal muscle, 189 861 for MTRFR deficiency, and 7216 for translation inhibition.

Table S1: The encoded 3' end of the CDS from mitochondrial mRNAs across primates and the house mouse.

Order	Genus species	*ND1*	*ND2*	*CO1*	*CO2*	*ATP8*	*ATP6*	*CO3*	*ND3*	*ND4L*	*ND4*	*ND5*	*CYTB*	*ND6*
Primates	*Homo sapiens*	UA	U	AGA	UAG	UAG	UAA	U	U	UAA	U	UAA	U	AGG
	Pan troglodytes	UA	U	AGA	UAG	UAG	UAA	U	U	UAA	U	UAA	U	AGG
	Macaca mulatta	UA	U	UAA	UAA	UAA	UAA	U	U	UAA	U	UAA	U	AGG
	Callithrix pygmaea	UA	U	AGG	U	UAA	UAA	U	U	UAA	U	UAA	UAA	UAA
	Tarsius dentatus	UAA	U	UAG	UAA	UAA	UAA	U	UA	UAA	U	UAA	UAA	UAA
	Microcebus berthae	UA	UAG	AGA	UAA	UAG	UAA	U	UAA	UAA	U	UAA	AGA	UAA
Rodentia	*Mus musculus (C57BL/6)*	U	U	UAA	UAA	UAA	UAA	U	UAA	UAA	U	UAA	U	UAA

The reference mitochondrial genome sequences: NC_012920; NC_001643.1; NC_005943.1; NC_021942.1; NC_024052.1; NC_035629.1; NC_005089.1.
